# Systems analysis-based assessment of post-treatment adverse events in lymphatic filariasis

**DOI:** 10.1371/journal.pntd.0007697

**Published:** 2019-09-26

**Authors:** Britt J. Andersen, Bruce A. Rosa, Jonah Kupritz, Aboulaye Meite, Traye Serge, Marla I. Hertz, Kurt Curtis, Christopher L. King, Makedonka Mitreva, Peter U. Fischer, Gary J. Weil

**Affiliations:** 1 Infectious Diseases Division, Department of Internal Medicine, Washington University School of Medicine, St. Louis, Missouri, United States of America; 2 McDonnell Genome Institute, Washington University School of Medicine, St.Louis, Missouri, United States of America; 3 Programme National de la Lutte Contre la Schistosomiase, Les Geohelminthiases et la Filariose Lymphatique, Abidjan, Côte d’Ivoire; 4 Center for Global Health and Diseases, Case Western Reserve University School of Medicine, Cleveland, Ohio, United States of America; Liverpool School of Tropical Medicine, UNITED KINGDOM

## Abstract

**Background:**

Lymphatic filariasis (LF) is a neglected tropical disease, and the Global Program to Eliminate LF delivers mass drug administration (MDA) to 500 million people every year. Adverse events (AEs) are common after LF treatment.

**Methodology/Principal findings:**

To better understand the pathogenesis of AEs, we studied LF-patients from a treatment trial. Plasma levels of many filarial antigens increased post-treatment in individuals with AEs, and this is consistent with parasite death. Circulating immune complexes were not elevated in these participants, and the classical complement cascade was not activated. Multiple cytokines increased after treatment in persons with AEs. A transcriptomic analysis was performed for nine individuals with moderate systemic AEs and nine matched controls. Differential gene expression analysis identified a significant transcriptional signature associated with post-treatment AEs; 744 genes were upregulated. The transcriptional signature was enriched for TLR and NF-κB signaling. Increased expression of seven out of the top eight genes upregulated in persons with AEs were validated by qRT-PCR, including *TLR2*.

**Conclusions/Significance:**

This is the first global study of changes in gene expression associated with AEs after treatment of lymphatic filariasis. Changes in cytokines were consistent with prior studies and with the RNAseq data. These results suggest that *Wolbachia* lipoprotein is involved in AE development, because it activates TLR2-TLR6 and downstream NF-κB. Additionally, LPS Binding Protein (LBP, which shuttles lipoproteins to TLR2) increased post-treatment in individuals with AEs. Improved understanding of the pathogenesis of AEs may lead to improved management, increased MDA compliance, and accelerated LF elimination.

## Author summary

Lymphatic filariasis (LF) is a disabling parasitic disease that affects millions of people in the developing world. The Global Programme to Eliminate Lymphatic Filariasis (coordinated by the World Health Organization) uses mass administration of antifilarial medications to cure infections, prevent disease, and reduce transmission. Some individuals develop adverse events (AEs) after treatment, and this can reduce willingness of persons in endemic areas to accept treatment. The purpose of this study was to improve understanding of the cause of AEs following treatment. We hypothesized that parasite antigens released into the blood following treatment trigger inflammatory responses that lead to AEs. To test this hypothesis we collected blood from LF-infected individuals before and after treatment and clinically assessed them for AEs. We measured parasite antigens, cytokines and other components of the immune system in blood samples and compared post-treatment changes in persons with and without AEs. We also assessed changes in transcription profiles in peripheral blood leukocytes that were associated with post-treatment AEs. Post-treatment changes in transcription profiles and in immune proteins and parasite components in plasma suggest that systemic AEs are triggered by death of the parasites following treatment with release of parasite antigens and *Wolbachia* bacteria into the circulation. Improved understanding of the pathogenesis of post-treatment AEs may help to improve messaging related to mass drug administration programs and lead to improved AE management.

## Introduction

Lymphatic filariasis (LF) is a disabling neglected tropical disease that is caused by the mosquito-borne filarial parasites *Wuchereria bancroft*i, *Brugia malayi* and *B*. *timori*. Adult worms live in the human host’s lymphatic system and release larval parasites (microfilariae or Mf) that circulate in the blood. Infection and host inflammatory responses to the parasite can lead to severe morbidity including lymphedema, hydrocele and elephantiasis [1]. To combat this disease the WHO launched the Global Program to Eliminate Lymphatic Filariasis (GPELF) in the year 2000 with the goal of eliminating LF as a public health problem by 2020. The program uses mass drug administration (MDA), to cure infections, prevent disease, and reduce transmission of new infections. As of 2016 a total of 6.7 billion treatments had been delivered to more than 850 million individuals [2], making GPELF the largest public health intervention for an infectious disease to date based on MDA. Drugs used for LF MDA include albendazole (ALB), ivermectin (IVM) and diethylcarbamazine (DEC). MDA with two-drug combinations is usually provided annually for 4–6 years. The combinations used are ALB with IVM in sub-Saharan Africa and ALB with DEC in other regions [1]. New studies have shown that combining all three drugs increases the anti-filarial effect and potentially decreases the number of required treatment rounds [3–7]. This new triple therapy (IDA) was recently recommended by the WHO as the preferred regimen for LF elimination in some settings [8].

Although LF treatment is safe, transient mild to moderate systemic adverse events (AEs) are common following treatment, and these are especially common in individuals with circulating Mf [3]. Furthermore, the risk of AEs and AE severity are positively correlated with blood Mf counts (Mf/mL) [9]. Systemic AEs are not direct effects of the drugs on the host, because they are quite uncommon in uninfected individuals [10]. The pathogenesis of these AEs is not completely understood, but they are believed to be trigged by host responses to dying filarial worms. Post-treatment AEs have been associated with increases in plasma levels of IL-6, TNF-α and soluble TNF receptor [11, 12]. We recently reported significant increases in 16 cytokines in persons who experienced AEs after treatment during a clinical trial that was performed in Papua New Guinea [13]. These results were consistent with LPS-like stimulation of cytokines with increases in TNF-α, IL-1β, IL-6, IL-1RA and IL-10.

*Wolbachia* are intracellular α-proteobacteria that are present in filarial species that cause LF. The bacteria are hypothesized to trigger AEs when they are released by dying parasites after treatment. One study detected free *Wolbachia* DNA in blood collected 4–48 hours after LF treatment in individuals with moderate and severe AEs, but bacterial DNA was not detected in blood from most individuals with no or mild AEs [14]. Some features of AEs are consistent with the effects of LPS. A filarial (*Brugia malayi*) antigen with LPS-like characteristics was described some years ago [15]. However, the *B*. *malayi*-associated *Wolbachia* genome [16] does not include orthologues of genes responsible for the biosynthesis of lipid A (a component of LPS) [17]. It is therefore unlikely that *B*. *malayi Wolbachia* contains LPS in its cell wall. Bioinformatic analysis of the *Wolbachia* genome predicts the presence of a *Wolbachia* lipoprotein: peptidoglycan-associated lipoprotein (PAL) [18]. A synthetic, lipolated version of the N-terminus of *Wolbachia* PAL can signal through TLR2-TLR6 and induce pro-inflammatory responses *in vitro* in murine and human cells and *in vivo* in mice [18]. Additionally, the diacylated N-terminal polypeptide of the *Wolbachia* PAL (WoLP) was identified as the main trigger for a neutrophil inflammatory response through a TLR2-TLR6 dependent mechanism *in vivo* in human samples from individuals infected with *Onchocerca volvulus* [19]. Recently PAL was confirmed by proteomics as one of the most abundant proteins in extracts from adult *B*. *malayi* female worms [20].

Besides *Wolbachia*, post-treatment AEs could also be triggered by immune complexes (IC) that develop after treatment of LF. ICs are aggregated antigens, antibodies, and components of the complement cascade that can activate pro-inflammatory pathways [21]. It has been reported that filarial antigen levels increase with a concurrent decrease in filarial specific antibodies post-treatment, and these changes were temporally associated with the development of AEs, suggesting that AEs might be caused by IC [22]. Circulating IC (CIC) have also been shown to increase post-treatment [23], and CIC precipitated from LF-infected individuals can activate granulocytes to release pro-inflammatory cytokines [24]. CIC activate the classical complement pathway.

AEs are common after treatment for LF, and fear of AEs reduces population compliance with MDA [25]. Therefore, the goal of this study was to improve understanding of the pathogenesis of AEs after LF treatment. We hypothesized that AEs are caused when filarial worm components are released after treatment and interact with the host innate or adaptive immune systems and that this would be associated with specific biomarker and gene expression profiles. To test this hypothesis we measured filarial antigen, CIC, LPS Binding Protein (LBP) and components of the complement cascade in plasma before and after treatment, and we studied host transcriptional responses and cytokine profiles in LF-infected individuals who experienced AEs after treatment.

## Methods

### Study design and sample collection

Buffy coat and plasma samples were collected during an open label filiariasis treatment study in the Agboville District in southeastern Côte d’Ivoire (Clinicaltrials.gov NCT # 02974049). Written informed consent was obtained from all participants. Adults with *W*. *bancrofti* microfilaremia were randomly assigned to one of four treatment arms (all oral medications): the standard LF treatment regimen for Côte d’Ivoire (200μg/kg IVM plus 400mg ALB), IDA: 200μg/kg IVM plus 6mg/kg DEC and 400mg ALB, a single 400 mg dose of ALB, or a single 800 mg dose of ALB. A subset of ninety-five individuals treated with either IVM/ALB, IDA or 400mg ALB had samples processed for use in the AE study described in this paper. We selected these individuals based on the availability of pre- and post-treatment samples and clinical AE data. Metadata of these 95 individuals is shown in S1 Table.

A physical examination was performed shortly before treatment, and vital signs were recorded. A review of systems (ROS) questionnaire was also completed to assess subjective symptoms prior to treatment. Venous blood (3 to 4 mL in EDTA) was collected immediately before participants received treatment. Participants were interviewed and examined the next day to assess AEs, and venous blood was collected approximately 24 hours after treatment. Blood samples were centrifuged within an hour of collection, and plasma was removed. The buffy coat (approximately 500μL) was carefully aspirated with a pipette and added to 1.8mL of RNAlater (Ambion, Foster City, CA). The plasma samples and buffy coat/RNAlater samples were stored at the study site at -20°C, shipped frozen, and later stored at -80°C.

### Adverse event classification

AEs were categorized as mild, or moderate. Those with moderate AEs (n = 9) had at least two new or worsening subjective symptoms plus one objectively measured change in their vital signs (an increase in axillary temperature of ≥ 0.8°C to at least 37.4°C post-treatment and/or a decrease in sitting systolic blood pressure of at least 20 mm Hg). Individuals with subjective or objective AEs that did not fulfill the criteria for moderate AEs were considered to have had mild AEs (n = 24). Individuals with no new objective or subjective symptoms after treatment were considered to have no AEs (n = 62).

### Circulating filarial antigen assay

A direct sandwich enzyme immunoassay (EIA) was performed as previously described [13]. This assay uses the monoclonal antibody AD12 that binds to a carbohydrate epitope on circulating filarial antigen (CFA). It is important to note that the carbohydrate epitope recognized by AD12 is present in many filarial glycoproteins [26]. However, the high molecular weight CFA is the only filarial antigen that is frequently detected in the blood of *W*. *bancrofti*-infected individuals. Pre- and post-treatment plasma samples from 95 individuals were tested in duplicate. The detection range of the CFA EIA was 6.3 to 400 ng/mL. CFA was detected in all samples, but two individuals had extremely high CFA levels that were above the upper detection limit of the assay. These samples were retested after dilution to obtain baseline CFA concentrations. The percent change in CFA relative to baseline following treatment was calculated for each participant. Sample pairs with pre-treatment values less than 20 ng/mL (7 individuals) were excluded from the percent calculations, because they were near the lower detection limit of the assay. Kruskal-Wallis H tests were used to compare percent change values and absolute values between the three AE groups and the three treatment arms. Wilcoxon signed-rank tests were used to compare pre- and post-treatment CFA levels within the three AE groups.

### Immunoprecipitation and Western blot

Nine paired (pre- and post-treatment) samples with high CFA levels at baseline were selected for this analysis; six of these participants had moderate AEs, one had mild AEs, and two had no AEs. 15mg of a monoclonal antibody (DH6.5) that detects the same carbohydrate epitope as AD12 was directly conjugated to 2mL of agarose Affigel 10 beads (Bio-Rad, Hercules, CA) according to the manufacturer’s protocol. Conjugated beads were stored as a 50% solution in PBS. 40μL of conjugated beads were mixed with 50μl of human plasma and 300μl PBS and rocked overnight at 4⁰C. The beads were washed four times with cold PBS and then boiled in 1X NuPAGE LDS sample buffer (Invitrogen, Carlsbad, CA) to release bound antigens. Proteins were resolved by SDS-PAGE using a 4–12% bis-tris NuPAGE gradient gel (Invitrogen) and transferred to 0.45μM nitrocellulose membrane (Amersham, Piscataway, NJ). Membranes were blocked with 5% milk in phosphate buffered saline with tween-20 (PBS-T) followed by incubation with a peroxidase-conjugated AD12 antibody (1:3000 dilution) for one hour at room temperature. After washing, membranes were incubated with Clarity Western ECL substrate (Bio-Rad). Chemiluminescence was detected by a ChemiDoc imager (Bio-Rad), and results were analyzed using Image Lab 5.2.1 software.

### Immune complex assay

CIC were measured with a C1q ELISA. C1q was purchased (Sigma-Aldrich, St. Louis, MO), and a previously published protocol was followed [13]. Plasma samples were available from 41 individuals for this assay (8 with moderate AEs, and 33 with no AEs), and both pre- and post-treatment samples were tested in duplicate. Negative control samples (plasma samples from healthy North American subjects and deionized water) were tested on each plate. Values were expressed as μg/mL of AHG (aggregated human gamma globulin) (Invitrogen). The range of detection for the CIC ELISA was 0.0006 to 6 μg/mL of AHG, and all samples had detectable CIC. Mann-Whitney U tests were used to compare absolute CIC levels between the two AE groups pre- and post-treatment. The Wilcoxon signed-rank test was used to compare post-treatment CIC levels to baseline levels within AE groups. The Kruskal-Wallis H test was used to compare absolute CIC levels between the three treatment arms post-treatment.

### Complement component assays

Nine individuals with moderate AEs were matched to individuals with no AEs following treatment. Matching was based on age, sex, baseline Mf count, and treatment arm (S2 Table). Complement component 3 (C3), complement component 4 (C4) and Factor B (FB) were measured in the 36 samples (18 matched case-control subjects pre- and post-treatment) with ELISA kits (AssayPro, St. Charles, MO). The C3 and C4 assays were competitive enzyme immunoassays, and the FB assay was a sandwich ELISA. Each sample was tested in duplicate and manufacturer’s protocol was followed. Paired t-tests were used to compare pre- and post-treatment complement component levels by AE group.

### LPS binding protein assay

LBP was measured with a sandwich ELISA kit (Abnova, Taipei, Taiwan). Plasma samples from the same 18 matched case-control subjects were included. Each sample was tested in duplicate and manufacturer’s protocol was followed. Paired t-tests were used to compare pre- and post-treatment levels within both AE groups. The range of detection for the LBP ELISA was 5 to 50 ng/mL.

### Cytokine assays

Twenty-seven cytokines (IL-1β, IL-1RA, IL-2, IL-4, IL-5, IL-6, IL-7, IL-8, IL-9, IL-10, IL-12 (p70), IL-13, IL-15, IL-17, basic FGF, eotaxin-1, G-CSF, GM-CSF, IFN-γ, IP-10, MCP-1, MIP-1α, MIP-1β, PDGF-BB, RANTES, TNF-α, and VEGF) were measured with the MAGPIX system with the Bio-Plex Human 27-Plex Cytokine Panel and Bio-Plex Cytokine Reagent Kit (Bio-Rad). Plasma samples from the same 18 matched-control subjects were included. A previous paper includes the detailed protocol [13]. Briefly, all samples were tested in duplicate, standard curves were calculated using the manufacturer’s software, and the analysis considered mean concentrations (pg/mL) from two duplicate wells. Wilcoxon signed-rank tests were used to determine whether cytokine levels changed after treatment in either of the two AE groups. Mann-Whitney U tests were used to determine whether pre- or post-treatment cytokine levels were different between the two AE groups. For graphing, fold changes were calculated for each cytokine by AE groups, and samples with cytokines below the detection limit were assigned a value equal to half the value of the lowest pre-treatment sample concentration measured for that cytokine.

### RNA preparation

RNA was extracted from pre- and post-treatment buffy coat samples from the same 18 matched case-control subjects. Total RNA was extracted using Qiagen RNeasy kits (Qiagen, Hilden, Germany) according to the manufacturer’s protocol with an added homogenization step and on-column DNase digestion as follows. For each sample 200μL of the buffy coat/RNAlater mixture was added to 700μL of the kit’s RLT buffer and vortexed. The mixture was then added to a QIAShredder column (Qiagen) and centrifuged for 2 min at 16,000g. The flow-through was added to 700μL of 70% ethanol, and this mixture was added to a RNeasy column. Bound RNA was eluted in 30μL RNase-free water and stored at -80°C. The quality and quantity of RNA was verified with a Bioanalyzer 2100 (Agilent Technologies, Cedar Creek, Texas). Samples were processed with the TruSeq Stranded Total RNA LT Sample Prep Kit with Ribo-Depletion using the manufacturer’s protocol (Illumina, San Diego, CA). The RNA was high quality (average RIN value 9.3, range 8.5–10).

### RNA sequencing and mapping

The 36 samples were sequenced in two batches. The first 14 samples were sequenced with the HiSeq2000 (2x 100 PE run and Illumina TruSeq Stranded Total RNA) platform, and the remaining 22 samples were sequenced with HiSeq4000 (2x 150 PE run and Illumina TruSeq Stranded Total RNA). Between 28–41 million read fragments per sample were mapped to 19,864 protein-coding genes. Raw reads were mapped to protein coding genes using HISAT2 (version 2.0.5) [27], and the human reference genome GRCh38.84. FeatureCounts [28] was used to count reads per gene.

### Differential gene expression and overall expression patters

DESeq2 [29] was used to generate normalized read counts and to identify differentially expressed genes between the different comparator groups (namely AEs vs. no AEs and pre- vs. post-treatment). The program “R” with the biocLite package “DESeq2” was used. Gene expression results from individuals before and after treatment were considered to be repeated measures for the analysis. Principal component analysis (PCA) was performed for 500 genes with the greatest variability in expression (based on DESeq2 output, default settings), and distance metrics statistics [30] were used to determine whether groupings affected overall expression patterns. A clustering dendrogram (Euclidean distance, complete linkage) was also used to illustrate overall expression patterns, and this method considered all genes. A two-tailed binomial distribution with unequal variance (for categorical data), and Mann-Whitney U tests (for continuous variables) were used to identify over-represented metadata variables in the different clustering groups such as baseline Mf/mL and treatment group.

### Functional enrichment in the post-treatment AE group

The online tool WebGestalt [31] was used to identify enriched KEGG pathways within genes that were upregulated post-treatment during AEs. The reference set was a list of all 19,864 genes with expression signals in the RNAseq data, and the default values were used except the significance level (FDR < 0.05). The program i-cisTarget [32] was used to identify enriched transcription factor binding sites in the upregulated gene set using default settings and database version 4.0.

### Identification of similar expression profiles

GeneQuery is an online tool that can search the PubMed GEO database and compare transcriptional signatures to published gene expression profiles [33]. The input for the post-treatment AE profile were 744 genes that were identified by differential gene expression (DESeq2) to be upregulated post-treatment in individuals with moderate AEs.

### Changes in peripheral blood leukocyte populations after treatment

CIBERSORT[34] is an analytical tool that can estimate the abundances of 22 leucocyte subtypes based on RNA-seq data. Pre- and post-treatment DESeq2 normalized read counts were used as input for the program. The standard LM22 (22 immune cell types) was the signature gene file, and all default settings were used. Thirteen cell subtypes had very low representation in this dataset (totaling less than 4% in all 36 samples), so the analysis was limited to the remaining subtypes. Percent change for each cell type post-treatment was calculated for the two AE groups, and Mann-Whitney U tests were used to assess the significance of differences by AE group.

### Prioritization of the genes with altered regulation post-treatment in individuals with AEs

Random forest (RF) analysis was used to prioritize the 678 genes that were differentially expressed in individuals with moderate post-treatment AEs. RF was performed using the “R” package “randomForest” with 1000 trees and default values to analyse DESeq2 normalized read counts. Differentially expressed genes were ranked based on decreasing Mean Decrease in Accuracy values for 10 separate RF models. The Mean Decrease in Accuracy is the decrease in model accuracy from permuting the values in each feature. This metric is used to compare the impact of the variables in the model, and a large positive value indicates that a variable was closely linked to AE group across the dataset.

### Preparation of cDNA for validation of selected differentially expressed genes

Additional RNA was extracted from residual buffy coat samples that were available for 34 of the 36 samples that were subjected to expression profiling (17 individuals pre- and post-treatment) as described above. Extracted RNA was treated with DNase I (Invitrogen), and RNA was measured with a NanoDrop 1000 Spectrophotometer (Thermo Scientific, Waltham, MA). Each sample was diluted to approximately 0.5ng/μL RNA with RNase-free water. cDNA was prepared with SuperScript II Reverse Transcriptase (Invitrogen) and with Oligo(dT)_12-18_ according to the manufacturer’s protocol_._

### Validation of the top differentially expressed genes by quantitative reverse-transcription PCR (qRT-PCR)

SYBR Green based assays were performed for the top eight genes based on the RF analysis (*DIP2B*, *ZCCHC6*, *RBPJ*, *PELI1*, *FNDC3B*, *TLR2*, *LTBR*, *NT5C2*) that were upregulated in peripheral blood leukocytes (PBL) after treatment in participants who experienced moderate AEs. Four housekeeping genes (*SDHA*, *ACTB*, *HPRT1* and *YWHAZ*) were used as controls for these experiments. We chose these based on prior validation as housekeeping genes by others [35] and because our results confirmed their stable gene expression before and after treatment. Pre-validated primer sets for the eight target genes were purchased from KiCqStart SYBR Green Primers (Sigma-Aldrich), and primers for the four housekeeping genes were made using previously published sequences (IDT, Coralville, IA) (S3 Table). Real-time PCR reactions were performed with 10μL of SYBR Green Master Mix (Applied Biosystems, Foster City, CA), 450 nmol/L of each primer, and 2μL cDNA (approx. 1ng RNA) with a final volume of 20μL. Thermal cycling was performed for 40 cycles with a QuantStudio 7-Plex Real-Time PCR System (Applied Biosystems), and cycle threshold (Ct) values were determined using the manufacturer’s software. All samples were tested in duplicate, and each plate included a negative water control and a RNA sample that had not been treated with reverse transcriptase. Delta delta Ct values were calculated [36], using the geometric mean Ct value of three housekeeping genes (*SDHA*, *ACTB* and *YWHAZ*) as a normalization factor [35]. Student’s t-tests were performed to compare baseline and post-treatment delta Ct values by AE group.

### Statistical methods

All statistical analyses were performed with IBM SPSS (version 23). Shapiro-Wilk tests were used to test for normality in each sample set, and additional tests were performed as described in each section above. Logistic regression analysis was performed with the binary dependent variable AEs (moderate AEs *vs*. no AEs). The independent variables considered included age, sex, treatment arm, baseline Mf/mL, and baseline CFA level.

Separate RF analyses of gene expression and plasma biomarker data were performed 10 times using 1000 trees. The output was the average Mean Decrease in Accuracy over the 10 runs for each variable.

### Ethical review

Institutional review boards in Cleveland, USA (University Hospitals Cleveland Medical Center IRB #08-14-13) and in Côte d’Ivoire (Comité National d’Ethique et de la Recherche, CNER, N: 008/MSLS/CNER/-kp) approved the clinical trial study protocol. Written informed consent was received from all participants prior to inclusion in the study.

## Results

### Study population

This study of the pathogenesis of AEs that occur after LF treatment used human samples that were obtained as part of a clinical trial for LF that was conducted in Côte d’Ivoire. Full results from that study have not yet been published, but early results from the study have been reported in published abstracts [4, 5]. Briefly, 189 *W*. *bancrofti*-infected adults were randomly assigned to one of four treatment arms as described above, and all participants had AE assessments performed 24 hr after treatment. The AE study enrolled a subset of 95 treated participants. S1 Table summarizes the specific analyses that were performed on samples from each of the 95 individuals. Nine of these participants experienced moderate AEs (Table 1), 24 had mild AEs, and 62 had no AEs. There was no difference in age or sex distribution between the three AE groups (S4 Table).

**Table 1:** Clinical characteristics of the nine individuals with moderate adverse events (AEs) after LF treatment.

ID	Treatment arm	Sex	Age	Mf/mL^A^	Objective findings^B^ (Pre and Post)^C^	Subjective AEs^D^
5	IVM/ALB	M	35	349	37.0–38.5	HA(1), fever(1)
6	IVM/ALB	M	60	700	120/70–100/60	HA(1), N/V(1)
7	IVM/DEC/ALB	F	32	264	35.7–38.0	Fever(2), joint pain(1)
8	IVM/DEC/ALB	M	48	660	36.7–38.2	HA(1), fever(1), rash(1), cough(1)
9	IVM/DEC/ALB	M	52	229	36.3–37.4	HA(2), fever(2), rash(1), joint pain(1), fatigue(1)
10	IVM/DEC/ALB	M	34	308	37.0–37.8	HA(1), fever(1), joint pain(1), muscle pain(1), fatigue(1)
11	IVM/ALB	M	29	560	37.5–38.5	Fever(1), rash(1), muscle pain(1)
12	IVM/DEC/ALB	M	69	503	170/90–120/80	N/V(1), rash(2), dark urine(1), cough(1), joint pain(1)
17	IVM/DEC/ALB	M	32	79	37.0–37.9	N/V(2), dyspnea(2)

^A^Mf/mL: microfilaria per mL of blood.

^B^Objective findings included an increase in axillary temperature of at least 0.8°C to at least 37.4°C post-treatment or a decrease in systolic blood pressure of at least 20mm post-treatment.

^C^Pre- and post-treatment temperatures or blood pressures are shown.

^D^Subjective AEs: symptoms not present at baseline that the patient reported during the post-treatment assessment. AE grading: 1, mild with no lost day at work or school; 2, the AE interfered with work or school attendance.

Abbreviations: HA, headache; N/V, nausea and/or vomiting.

### Multiple additional filarial antigens were detected in post-treatment plasma samples

**Fig 1:**
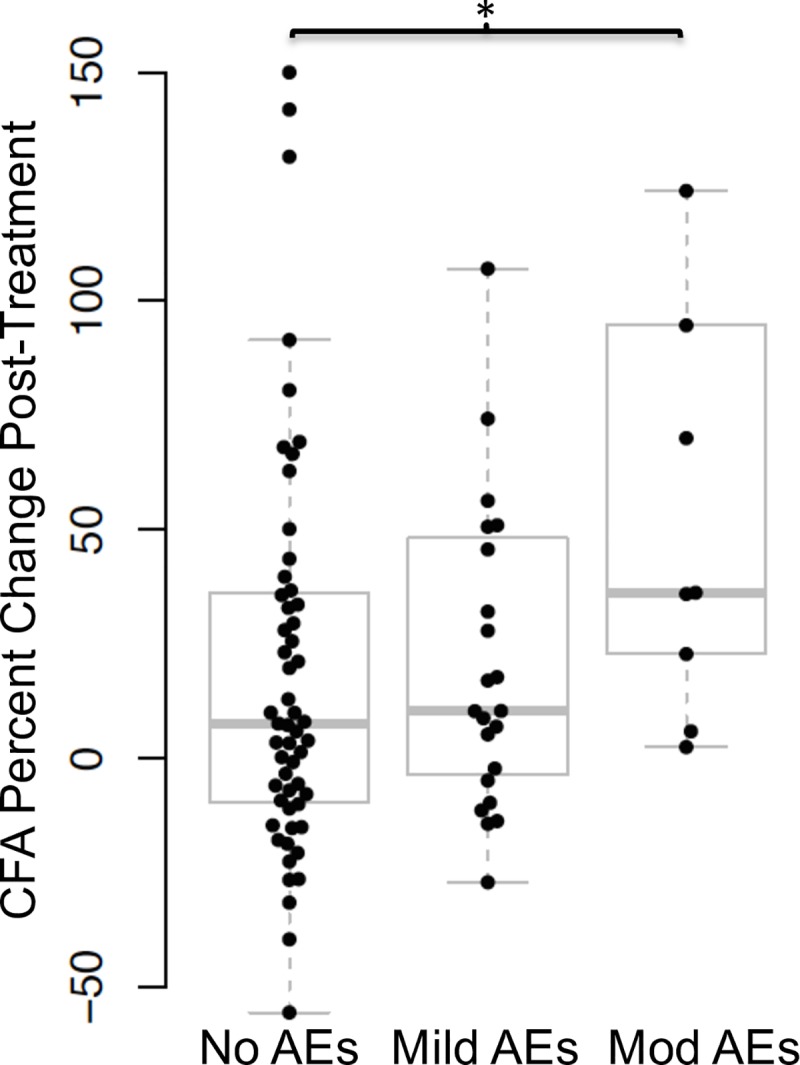
Filarial antigens increase post-treatment in individuals with moderate adverse events (AEs). Circulating filarial antigen (CFA) levels increased significantly more post-treatment in individuals who experienced moderate adverse events (mod AEs) compared to individuals with no AEs (*P* < 0.05 by Kruskal-Wallis) (n = 62 with no AEs, n = 24 with mild AEs, n = 9 with moderate AEs). Boxes indicate the interquartile range (25^th^ and 75^th^ percentile of data distribution), and horizontal lines within the boxes are median values. The whiskers show 95% confidence intervals around the median values.

Baseline CFA levels were positively correlated with baseline Mf counts (Spearman’s rho: 0.51, *P* < 0.001), and absolute CFA levels were significantly higher at baseline in individuals who developed moderate AEs compared to individuals who developed mild or no AEs after treatment (*P* = 0.012 by Kruskal-Wallis H test). Plasma CFA levels increased post-treatment in all three AE groups, but the increases were greater in persons with moderate AEs (*P* < 0.05 by Kruskal-Wallis H test) (Fig 1). Percent changes in CFA levels post-treatment were significantly lower in the individuals treated with only ALB compared to those in individuals treated with IVM/ALB or IDA (*P* < 0.0001 by Kruskal-Wallis H test).

Western blot analysis was performed for nine pre- and post-treatment plasma pairs to compare CFA patterns detected in plasma before and after treatment. All nine pre-treatment plasma samples contained only a single high molecular weight parasite antigen as expected, and this antigen was also present in post-treatment plasma samples. However, four of the post-treatment plasma samples contained many parasite antigens that were not present before treatment. Two examples are shown in S1 Fig (P1 had moderate AEs, and P2 had mild AEs), and this pattern was also observed in plasma from two other participants who experienced moderate AEs following treatment. Western blot results obtained with five other post-treatment plasma samples tested (3 from persons with moderate AEs, and 2 from persons with no AEs) were no different from those observed in pre-treatment samples.

### CIC did not increase post-treatment and the classical complement pathway was not activated in individuals with moderate AEs

All pre- and post-treatment samples contained CIC. However, there was no difference in CIC levels between the two AE groups before or after treatment, and CIC levels did not significantly change after treatment in either AE group (S2 Fig). There was also no difference in post-treatment CIC levels between the treatment arms.

**Fig 2:**
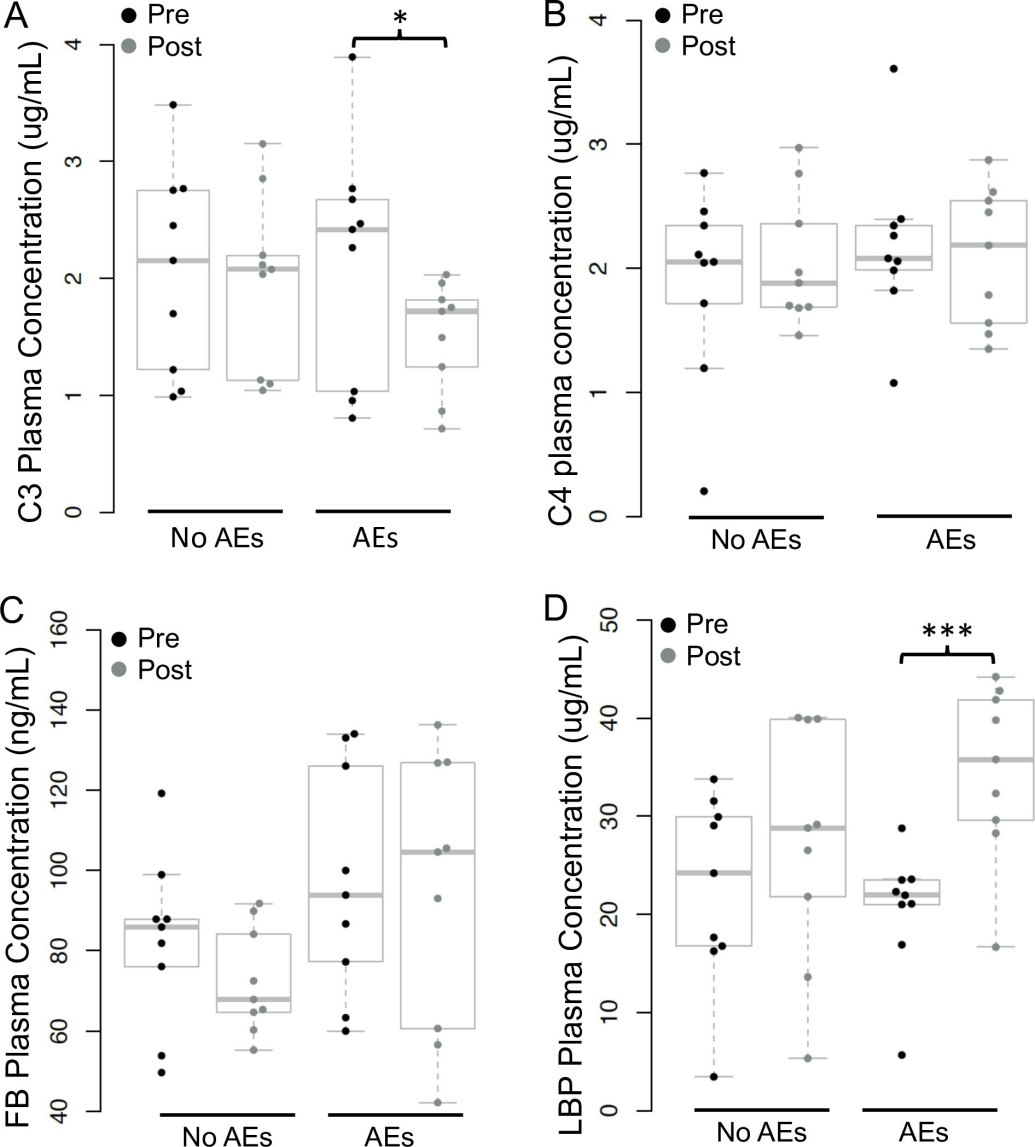
Levels of complement components and lipopolysaccharide binding protein (LBP) pre- and post-treatment (n = 9 with moderate adverse events (AEs), n = 9 with no AEs). Fig 2A: Complement component 3 (C3) significantly decreased post-treatment in individuals with moderate AEs (*P = 0.03 by paired t-test). B: Complement component 4 (C4) did not change with treatment in either AE group. C: Complement Factor B (FB) did not change with treatment in either AE group. D: LBP levels significantly increased post-treatment in individuals with moderate AEs (***P = 0.00007 by paired t-test).

C3 levels significantly decreased post-treatment in individual with moderate AEs, but this change was not observed in individuals with no AEs (Fig 2A). C4 levels did not change in either AE group (Fig 2B). Factor B levels decreased post-treatment in most individuals with moderate AEs, but three individuals had increases in FB levels, and the group differences were not significant (Fig 2C).

### LPS binding protein levels increased post-treatment in individuals with moderate AEs

LBP was detected in all pre- and post-treatment samples. LBP levels increased post-treatment in individuals with moderate AEs (*P* = 0.0007 by paired t-test), but they did not increase in individuals with no AEs (Fig 2D).

### Many plasma cytokines increased in plasma after treatment in persons who experienced moderate AEs

**Fig 3:**
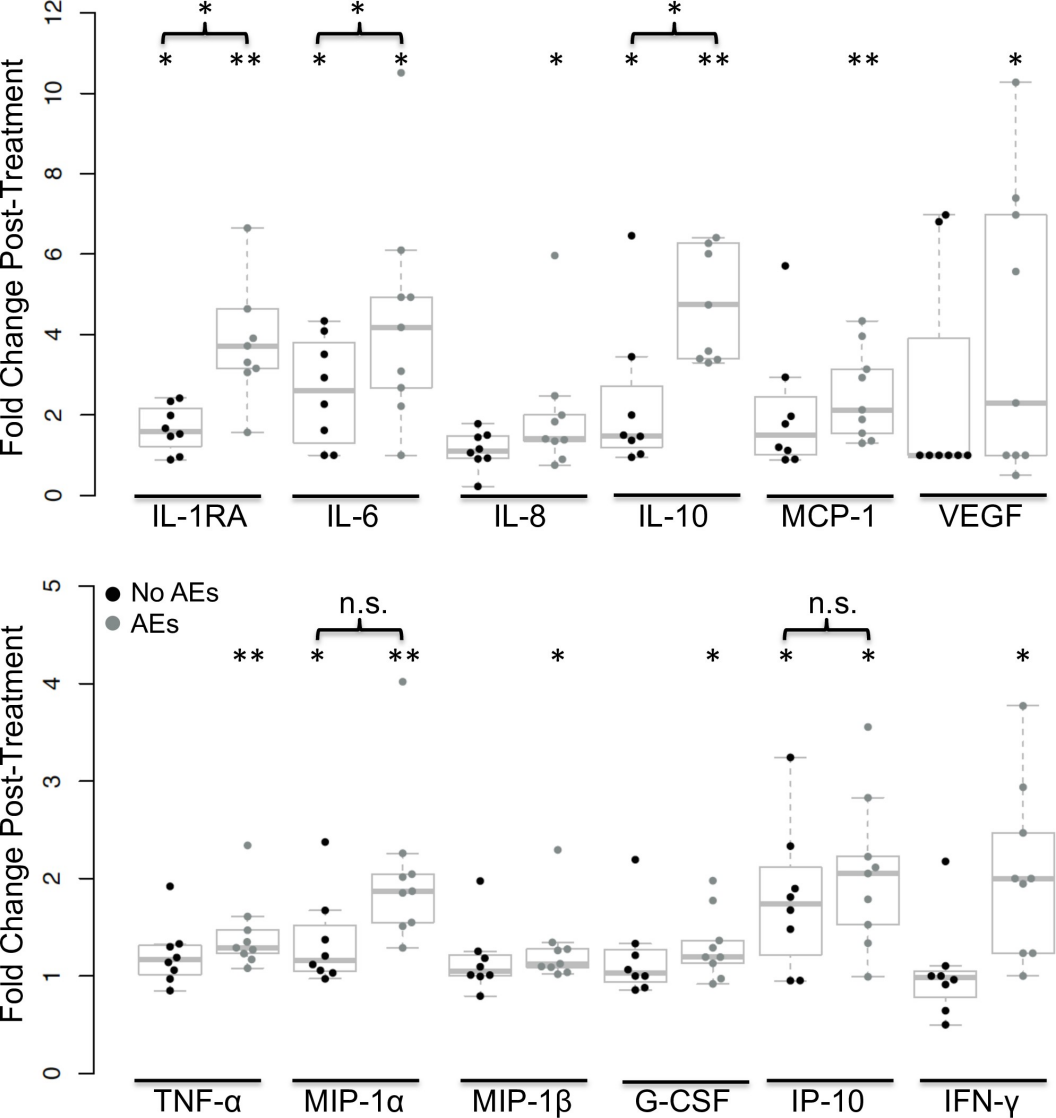
Post-treatment fold changes for 12 cytokines in nine participants who experienced moderate AEs and in nine participants who had no AEs. Levels of IL-8, MCP-1, VEGF, TNF-α, MIP-1β, G-CSF and IFN-γ significantly increased post-treatment in individuals with moderate AEs, and levels of IL-1RA, IL-6, IL-10, MIP-1α and IP-10 increased significantly post-treatment in individuals with moderate AEs and in individuals with no AEs (*P < 0.05, **P < 0.001 by Wilcoxon signed-rank tests). IL-1RA, IL-6 and IL-10 increased more in individuals with moderate AEs (*P < 0.05 by Mann-Whitney U tests).

Plasma cytokine levels before and after treatment are shown by AE group in Fig 3. Seven cytokines (IL-8, MCP-1, VEGF, TNF-α, MIP-1β, G-CSF and IFN-γ) increased post-treatment only in individuals who experienced moderate AEs (*P* < 0.05 by Wilcoxon signed-rank test). Five cytokines (IL-6, IL-10, IL-1RA, IP-10 and MIP-1α) increased post-treatment in individuals with and without AEs (*P* < 0.05 by Wilcoxon signed-rank test), but three of these (IL-6, IL-10 and IL-1RA) had significantly higher levels post-treatment in individuals with moderate AEs compared to individuals with no AEs (*P* < 0.05 by Mann-Whitney U tests) (Fig 3). The remaining 15 cytokines (IL-1β, IL-2, IL-4, IL-5, IL-7, IL-9, IL-12 (p70), IL-13, IL-15, IL-17, basic FGF, eotaxin-1, GM-CSF, PDGF-BB and RANTES) did not change after treatment in either AE group. There was no difference in pre-treatment cytokine levels between individuals that would develop moderate AEs and individuals that would not develop AEs for any of the 27 cytokines.

### Changes in gene expression associated with moderate post-treatment AEs

Raw and processed RNA-seq data are available to the public on NCBI’s Gene Expression Omnibus (Accession number: GSE110146).

**Fig 4:**
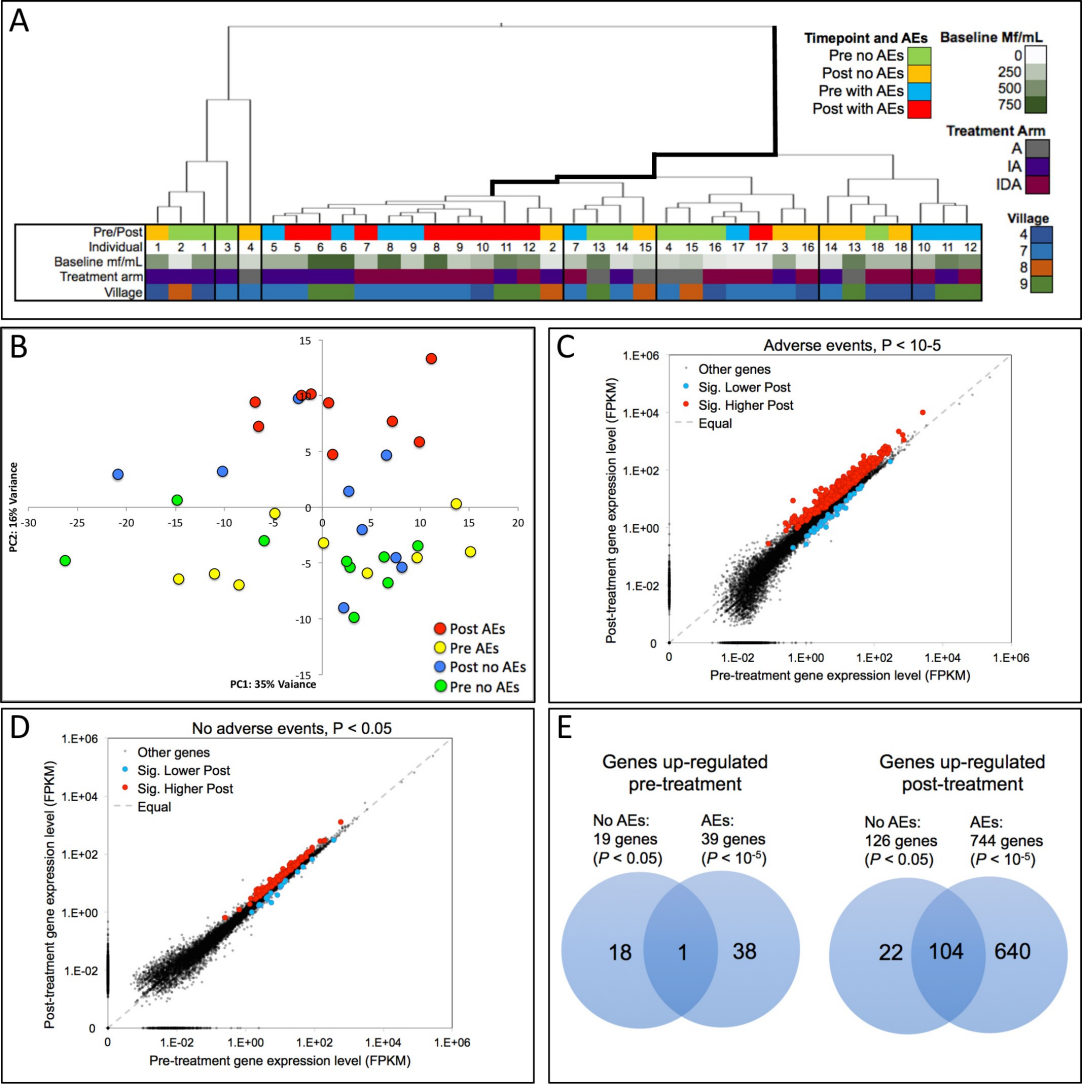
Overall expression patterns and enrichment pre- and post-treatment (n = 9 with moderate adverse events (AEs), n = 9 with no AEs). Fig 4A: Sample clustering (Euclidean distance) based on gene expression profiles across all genes. Post-treatment AEs (red) samples are significantly overrepresented in the fourth group (bolded, *P* < 0.0001, binomial distribution). Treatment arms; A: albendazole (ALB), IA: ivermectin (IVM) and ALB, IDA: IVM, diethylcarbamazine and ALB. B: Principal component analysis of paired samples. Post-treatment AEs samples (red) are significantly different from their pre-treatment controls (yellow) (*P* = 0.005 by PERMANOVA). No other differences between the four groups are significant. C/D: Expression plots comparing pre- and post-treatment gene expression in individuals with moderate AEs (C) and individuals with no AEs (D). Red dots represent genes significantly upregulated post-treatment, and blue dots are genes significantly upregulated pre-treatment (*P* < 10^−5^ for the AE group (C), and *P* < 0.05 for the no AE group (D) from DESeq2). E: Overlap between the genes upregulated pre- and post-treatment between the two AE groups.

We analyzed changes in gene expression in PBL after treatment to further elucidate host responses associated with AEs. Post-treatment gene expression profiles from persons who developed moderate AEs clustered together using a clustering dendrogram based on gene expression profiles across all genes (Fig 4A). Post-treatment AE samples were significantly overrepresented in the fourth group (bolded in Fig 4A, *P*-value < 0.0001 for enrichment within the cluster, two-tailed binomial distribution with unequal variance). Higher levels of baseline Mf/mL were also observed in this group (*P*-value = 0.038, Mann-Whitney U test), but none of the other metadata categories (treatment arm or village) were over-represented. Age also did not affect clustering. A similar pattern was observed by principal components analysis (Fig 4B), where post-treatment moderate AE samples clustered together and were clearly separated from their pre-treatment controls (*P*-value = 0.005 by PERMANOVA [30]). No other differences were significant between the four groups by PCA.

We used differential gene expression analysis to identify the genes that were responsible for the clustering of the post-treatment moderate AE samples. At a very stringent significance threshold (*P* < 10^−5^ according to DESeq2 output), 783 genes were identified to be upregulated after treatment (n = 744) or before treatment (n = 39) in individuals who experienced moderate AEs (Fig 4C). No differences were observed pre- or post-treatment in individuals with no AEs when this stringent significance threshold was used. However, at a less stringent *P*-value of 0.05, there were 126 genes upregulated post-treatment and 19 genes upregulated pre-treatment in individuals without AEs (Fig 4D). There was only one overlapping gene in the genes upregulated pre-treatment in individuals with and without AEs, whereas the majority of the genes upregulated post-treatment in individuals with no AEs were also upregulated post-treatment in individuals with AEs (Fig 4E).

We then assessed whether there was evidence for functional enrichment in the genes upregulated post-treatment in individuals with AEs. Among the 744 upregulated genes post-treatment in the AE samples a total of 35 enriched biological pathways (KEGG) were identified (S5 Table), and these included TLR signaling and downstream pathways such as NF-κB, TNF and Jak/STAT. Many individual genes in the TLR signaling pathway, including *TLR2*, *TLR6*, *STAT1* and *STAT2*, were identified by DESeq2 to be significantly upregulated post-treatment in individuals with AEs. A separate analysis (i-cisTarget) predicted that six transcription factors were over-represented in the differentially expressed genes (S6 Table), and three of these, *STAT1*, *STAT2* and *IRF1*, are downstream of TLR signaling. Convincingly, *STAT1* and *STAT2* were therefore identified by two independent analyses, signifying the importance of these two transcription factors in the development of AEs. IRF1 is activated by IFN-γ and is a major transcription factor of IL-8, and correspondingly both IFN-γ and IL-8 levels significantly increased post-treatment in individuals with AEs. The complete TLR signaling pathway highlighting the individual upregulated genes, KEGG pathways and transcription factors, was constructed with the use of the online database SPIKE [37] (S3 Fig). Finally we wanted to compare our newly identified LF AE transcriptional signature to published gene expression profiles. The post-treatment AE transcriptional signature of the 744 upregulated genes was very similar to multiple published endotoxin exposure gene expression profiles, in addition to many other profiles (S7 Table).

After successfully identifying a significant transcriptional signature of post-treatment AEs, we explored whether a pre-treatment transcriptional signature could predict what individuals would go on to develop AEs after treatment. There were no significant differentially expressed genes at baseline between individuals that would develop moderate AEs, and individuals that would not develop AEs (by DESeq2).

### Neutrophils increased and lymphocytes decreased post-treatment

**Fig 5:**
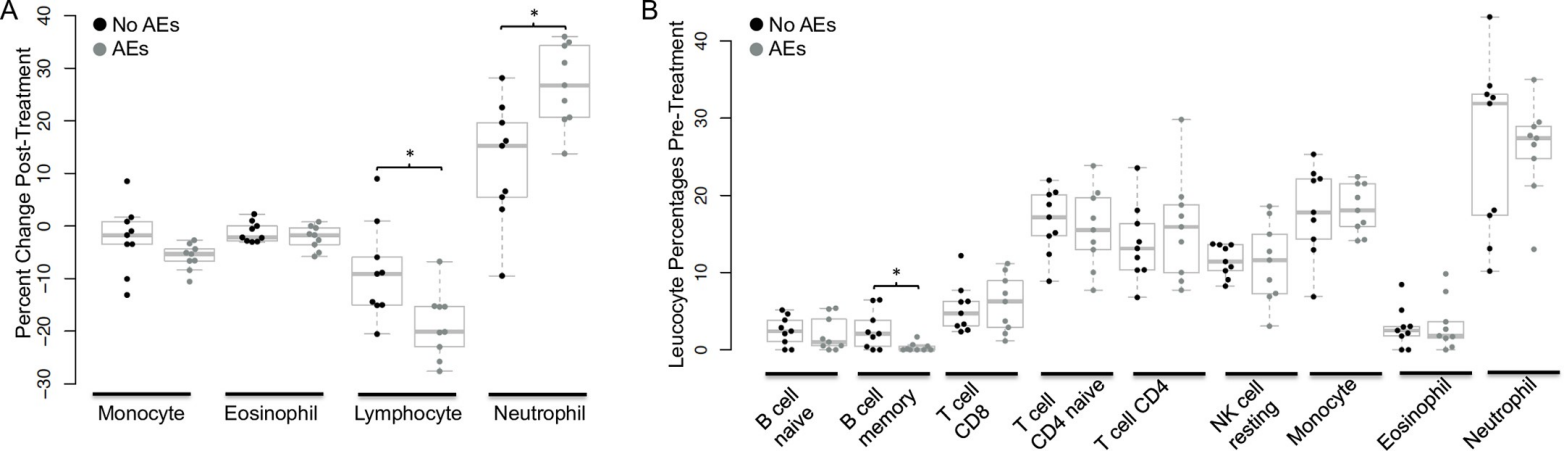
Estimated leucocyte subtypes (CIBERSORT) for the two adverse events (AEs) groups. Fig 5A: Percent change post-treatment of leukocyte subtypes. Estimated lymphocytes decreased more and estimated neutrophils increased more post-treatment in individuals with moderate AEs (n = 9) compared to individuals with no AEs (n = 9). B: Estimated leukocyte proportions pre-treatment. Individuals that did not develop AEs (n = 9) had higher levels of estimated memory B cells pre-treatment compared to individuals that developed moderate AEs (n = 9). *P < 0.05 by Mann-Whitney U tests.

Changing cell populations can have a large effect on gene expression profiles. Differential cell counts were unavailable, so cell type proportions were estimated using the RNA-seq data. This analysis suggested that neutrophils increased more and lymphocytes decreased more post-treatment in individuals with AEs compared to individuals with no AEs (Fig 5A). These changes are consistent with stress-type immune responses. For simplicity, B cells (memory and naïve), T cells (CD8, CD4 naïve and memory resting) and NK cells were combined into one category (lymphocytes) in Fig 5A, and ungrouped data are presented in S4 Fig. Estimated leucocyte proportions at baseline were very similar between the two AE groups, but individuals who experienced post-treatment AEs had significantly fewer estimated memory B cells compared to individuals who did not develop AEs after treatment (Fig 5B).

### Prioritization of genes upregulated post-treatment in individuals with AEs show that TLR2 is one of the most important genes for the development of AEs

The genes upregulated post-treatment in individuals with AEs were prioritized by importance for AE development using a machine-learning tool (RF analysis). This was done in order to identify genes with the strongest associations between expression levels and development of AEs and to identify genes of interest for PCR validation. Table 2 shows the top 15 genes that were upregulated in persons who developed moderate AEs after treatment. However, based on this analysis it was not possible to determine whether the gene expression changes were the cause or effect of the AEs that were experienced.

**Table 2:** Top 15 genes associated with development of moderate adverse events in LF patients after treatment as identified by random forest analysis.

Gene	Gene Description	Mean Decrease in Accuracy
*DIP2B*	Disco interacting protein 2 homolog B	3.30
*ZCCHC6*	Zinc finger CCHC-type containing 6	3.18
*RBPJ*	Recombination signal binding protein for Ig kappa J region	3.07
*PELI1*	Pelino E3 ubiquitin protein ligase 1	2.99
*FNDC3B*	Fibronectin type III domain containing 3B	2.95
*TLR2*	Toll like receptor 2	2.79
*LTBR*	Lymphotoxin beta receptor	2.65
*NT5C2*	5’-nucleotidase, cytosolic II	2.57
*KIAA1551*	KIAA1551	2.32
*ALDH1A2*	Aldehyde dehydrogenase 1 family member A2	2.08
*ZSWIM6*	Zinc finger SWIM-type containing 6	1.97
*APLP2*	Amyloid beta precursor like protein 2	1.95
*MYLIP*	Myosin regulatory light chain interacting protein	1.95
*FGD4*	FYVE, RhoGEF and PH domain containing 4	1.92
*IL1RAP*	Interleukin 1 receptor accessory protein like 2	1.90

### Orthogonal validation of expression levels for candidate genes confirmed RNA-seq data

**Fig 6:**
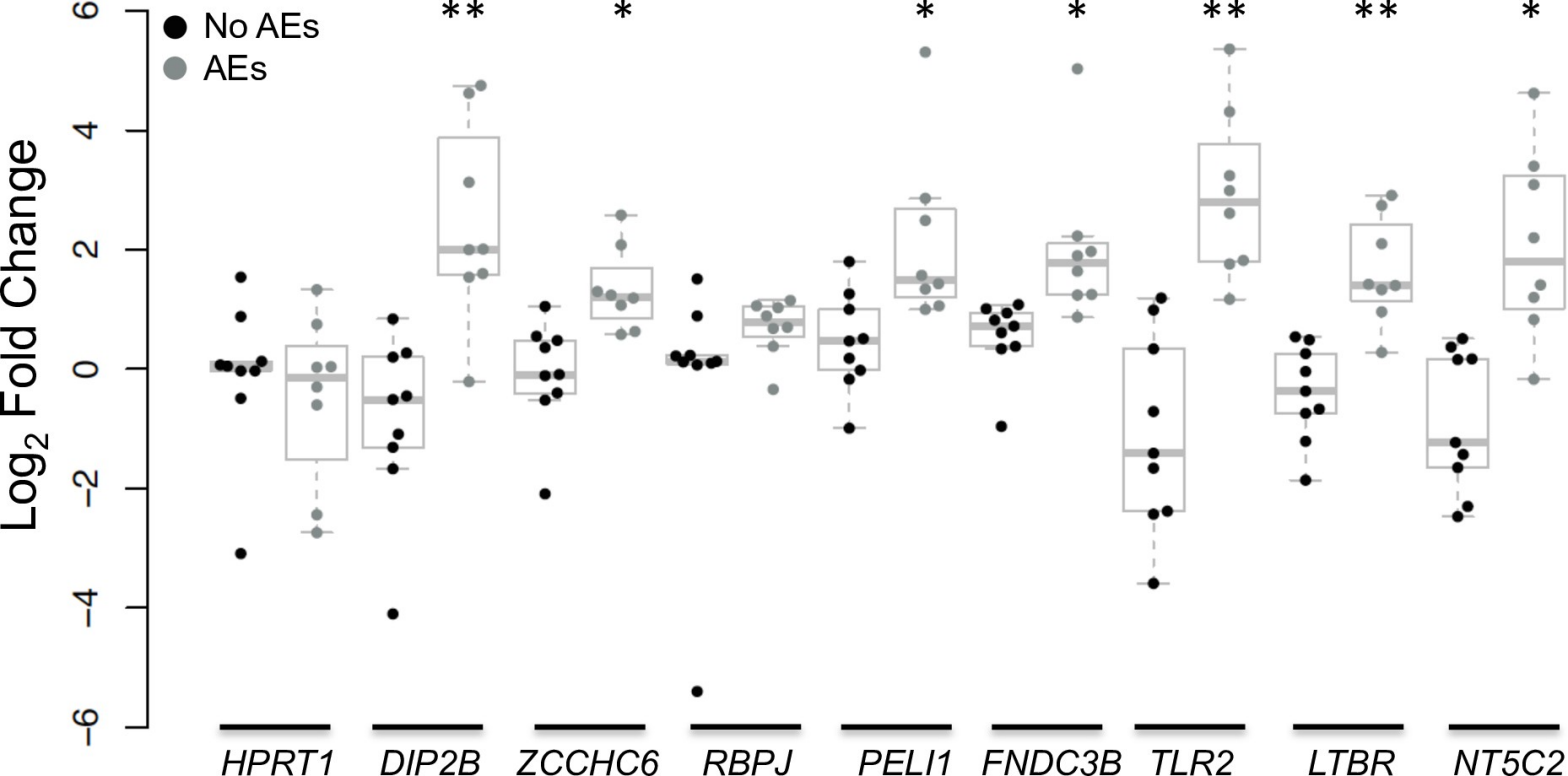
qRT-PCR validation of RNA-seq data. Post-treatment fold change of the top eight genes identified by random forest analysis. Seven of the genes (*DIP2B*, *ZCCHC6*, *PELI1*, *FNDC3B*, *TLR2*, *LTBR* and *NT5C2*) significantly increase only in individuals with moderate adverse events (AEs) (n = 8) compared to individuals with no AEs (n = 9). *P < 0.05, **P < 0.001 by t-tests. *HPRT1* is a housekeeping gene that should not change with treatment in either AE group.

qRT-PCR studies were performed to confirm whether expression of genes identified by DESeq2 and RF analyses was actually increased post-treatment in individuals with moderate AEs. Increased expression after treatment was confirmed for seven of the top eight genes (*DIP2B*, *ZCCHC6*, *PELI1*, *FNDC3B*, *TLR2*, *LTBR* and *NT5C2*) (Fig 6). Expression of the eighth gene (*RBPJ*) did not change after treatment in either AE group. Expression of the housekeeping gene *HPRT1*, did not change with treatment in either AE group (as expected).

### Modeling identified high baseline CFA levels as predictor for development of post-treatment AEs and post-treatment increases in LBP levels as another risk factor

We were unable to identify any pre-treatment transcriptional signature that could predict moderate AEs. We therefore wanted to assess if any metadata or baseline infection parameter was associated with the development of moderate AEs. A logistical regression was performed to consider effects of age, sex, treatment arm, baseline Mf/mL and baseline CFA on the risk for development of post-treatment moderate AEs. A total of 71 individuals were included in the model (9 moderate AEs and 62 no AEs). The logistic regression model was statistically significant, *X*^*2*^ (6) = 22.1, *P* = 0.0012. The model explained 50.2% (Nagelkerke *R*^*2*^) of the variance in AE outcome, and correctly predicted 93% of outcomes. However the model was better at predicting people who did not develop AEs; it correctly predicted only 44.4% of the individuals who developed moderate AEs. Increasing baseline CFA levels were associated with increased likelihood of developing AEs (*P* = 0.022), but the other independent variables did not significantly contribute to the model. RF analysis was performed on the same dataset (71 individuals), and this also identified the baseline CFA level as the best predictor for subsequent development of AEs. However, treatment arm was also a positive predictor in the RF model, and could therefore be related with the development of AEs (Table 3). It was surprising that baseline Mf count was not identified by the logistic regression model or RF to significantly contribute to correctly predicting AEs. However, baseline Mf counts were higher in individuals who developed moderate AEs (geometric mean 343 Mf/mL) compared to individuals with no AEs (geometric mean 188 Mf/mL), and this difference was significant (*P =* 0.036 by Mann-Whitney U test). RF analysis was also used to identify the variable (CFA, CIC, C3, C4, FB or LBP) that was best at classifying AE outcome based on post-treatment fold change in the 18 matched case-control subjects. LBP changes after treatment was the only variable that was significantly associated with the development of AEs (S8 Table).

**Table 3:** Logistic regression and random forest analysis of sex, age, treatment arm and baseline infection parameters on the development of moderate adverse events.

	Logistic Regression Model			Random Forest
Variable	*B*	Odds ratio	*P*-value	Mean Decrease in Accuracy
Constant	-4.79	-	0.006	-
Sex	-0.24	0.79	0.878	-2.82
Age	0.03	1.03	0.390	-0.94
Treatment arm^A^: IDA ALB	0.54 -19.28	1.72 0.00	0.558 0.998	4.33
Mf/mL	0.00	1.00	0.753	-2.82
CFA	0.02	2.69^B^	0.022	11.57

^A^The coefficients for treatment arms are contrasts with the standard treatment, ivermectin (IVM) plus albendazole (ALB). IDA: IVM, diethylcarbamazine (DEC) and ALB.

^B^Reported odds ratio for CFA (circulating filarial antigen) is for an increase of 50 ng/mL, because the range of CFA concentrations was wide (7–700 ng/mL).

## Discussion

This study looked at changes in proteins in plasma and changes in gene expression in PBL in persons who experienced moderate AEs following treatment of LF.

### Changes in proteins in plasma in persons who experienced AEs after treatment

Filarial antigen levels increased in plasma after treatment in individuals with moderate AEs, and this agreed with our recently published results from a separate clinical trial [13]. Western blot results from this study showed that many new filarial antigens with the carbohydrate epitope detected by the monoclonal antibody AD12 appeared in the blood 24 hours after treatment in some individuals. In contrast, only a single high molecular weight antigen circulates in the blood of *W*. *bancrofti*-infected individuals without treatment [26]. We postulate that treatment kills or injures worms so that they release internal antigens that are normally concealed inside the parasite.

Results from this study also confirmed our previous finding that plasma CIC levels do not increase after treatment of LF in persons who develop moderate AEs [13]. This finding suggests that AEs after LF treatment are not caused by CIC. The complement cascade (classical pathway) modulates pro-inflammatory effects of CIC [38]. Activation of the classical complement cascade leads to decreased C3 and C4, whereas activation of the alternative pathway (AP) leads to decreased C3 and factor B (FB). Our results are most consistent with activation of the AP by parasite antigens, as C3 and FB decreased in individuals with moderate AEs while there was no change in C4 levels. The RNA-seq data also supports the AP hypothesis, because expression of *CFP* (complement factor properdin- a positive regulator and initiator of the AP) significantly increased post-treatment in individuals with moderate AEs (adjusted *P*-value 0.001, DESeq2). In contrast, expression of *C4B* (basic form of C4, part of the classical pathway) significantly decreased (adjusted *P*-value 0.04, DESeq2) post-treatment in individuals with moderate AEs. Additionally, IFN-γ and TNF-α are known to induce FB synthesis [39]. This could account for the inconsistent FB levels post-treatment in individuals with moderate AEs, because both IFN-γ and TNF-α increased in these individuals; the positive stimulus of these cytokines may have counteracted decreases in FB levels as it was used in the AP. In summary, many different filarial antigens were transiently released post-treatment, but they did not appear to form CIC or activate the classical complement cascade.

PBL appeared to respond to the release of filarial antigens by releasing cytokines, and the cytokine profiles of post-treatment AEs in LF infected individuals are complex. In our previous study of samples from a treatment trial in Papua New Guinea, we reported that 16 cytokines increased post-treatment in individuals with moderate AEs [13], and eight of these cytokines were also increased after treatment in this study (IL-1RA, IL-6, IL-10, G-CSF, MCP-1, MIP-1β, TNF-α, and VEGF). It is not surprising that more cytokines increased in the previous study, because more time-points were sampled. Also, participants in the Papua New Guinea study had higher blood Mf counts and higher rates and severity of AEs than participants in the present study, and this may account for their stronger cytokine responses. A new finding in this study was the increase in IFN-γ post-treatment in individuals with moderate AEs. This was supported by the fact that IRF1 (downstream of IFN-γ) was identified to be an important transcription factor for AE development. The increase in IL-8 levels paralleled the increase in neutrophils post-treatment in individuals with moderate AEs. Results from this study did not confirm the finding from the prior Papua New Guinea study that high levels of eotaxin-1 pre-treatment are a risk factor for development of post-treatment AEs. Again this discrepancy may be related to differences in infection intensity between participants in the two studies. Levels of IL-6 and TNF-α have previously been shown to be positively correlate to levels of *Wolbachia* DNA in human plasma 48 hours after treatment of LF [40].

### Changes in gene expression in PBL in persons who experienced moderate AEs after treatment

RNA-seq was performed to better understand changes in leukocyte gene expression that occur in persons who experienced moderate AEs after treatment. We identified a distinctive transcriptional signature associated post-treatment moderate AEs with 783 genes that were differentially expressed (at the *P* < 10^−5^ level) in persons who experienced moderate AEs. In contrast, no gene was differentially expressed at that level of significance before or after treatment in individuals who did not experience AEs. 95% of the 783 genes associated with AEs exhibited increased expression post-treatment. Thus, moderate AEs were primarily associated with upregulation of gene expression and not with gene suppression. A total of 126 genes were upregulated post-treatment in individuals with no AEs at the low stringency *P* < 0.05 level, but 83% of these genes were also upregulated post-treatment in individuals with moderate AEs. Thus changes in gene expression after treatment did not always lead to clinically evident AEs.

The transcriptional signature results are consistent with the hypothesis that *Wolbachia* lipoprotein activates TLR2-TLR6 [18, 41], as bacterial lipoproteins can induce pro-inflammatory responses through TLR2 signaling and NF-κB and STAT1 activation [42]. The finding that *TLR2* was one of the genes most highly associated with the development of moderate AEs also supports this hypothesis. Furthermore, LBP was found to increase post-treatment in plasma from individuals with moderate AEs, and RF analysis identified LBP (fold change post-treatment) as the best variable for classifying AE outcome. LBP is an acute-phase protein that is mostly known for its function of shuttling LPS to TLR4 via CD14. However, it can also shuttle lipoproteins to TLR2 also via CD14 [43] as would be the case with PAL. *CD14* expression was upregulated post-treatment in individuals with moderate AEs, and it had one of the most significant adjusted *P*-values (6.4e^-26^) in the dataset. CD36 is another accessory receptor for the TLR2-TLR6 heterodimer [44], and this gene was also upregulated post-treatment in individuals with moderate AEs (adjusted *P*-value 0.004). The LF AE transcriptional signature was similar to previously published endotoxin exposure gene expression profiles further supporting the *Wolbachia* lipoprotein hypothesis. Since multiple filarial antigens that are normally not accessible to the immune system are released post-treatment, it is possible that *Wolbachia* components are released into the host’s circulation in a similar fashion. Thus we cannot be certain that *Wolbachia* lipoprotein is the only or prime trigger for AEs. It is possible that other filarial components can signal through TLRs and contribute to the development of AEs, and this might explain why expression of many other TLRs (*TLR1*, *TLR4*, *TLR5*, *TLR6* and *TLR8)* were upregulated post-treatment in individuals with moderate AEs. Many different ligands can activate TLR2, including protozoan ligands such as GPI anchors from *Trypanosoma cruzi* [45] and *Leishmania major* [46]. *Wolbachia*-independent activation of the immune system causing severe AEs is seen in *Loa loa (*a filarial worm that lacks *Wolbachia* [1]) infected individuals post-treatment suggesting that *Wolbachia* is not the sole cause of AEs after anti-filarial treatment. TLR signaling is clearly associated with the development of AEs, but complement activation has similar downstream effects, and there is considerable crosstalk between these two pathways [47]. Thus both TLR signaling and the complement AP could be actively involved in the pathogenesis of AEs. Another possible mechanism for AEs following treatment of LF is that treatment abrogates the normally dominant Th2 immune responses stimulated by helminth infections that interfere with the expression and function of TLRs [48]. Increased TLR expression and signaling after treatment may then induce pro-inflammatory Th1 responses causing AEs. Indeed, classic Th1 cytokines TNF-α and IFN-γ were increased after treatment in people with moderate AEs.

We did not detect a pre-treatment transcriptional signature that was a significant risk factor for development of post-treatment AEs. Baseline CFA levels were the best predictor for moderate AEs in this study, and this was the only variable that was a significant predictor by logistic regression. CFA levels are correlated with Mf counts and likely with adult worm numbers, and this result suggests that CFA levels are related to the total parasite biomass that can potentially contribute to the development of AEs after treatment.

### Genes upregulated post-treatment in individuals with AEs

An important finding from this study was that post-treatment AEs in LF-infected individuals are associated with upregulation of hundreds of genes. A prioritized list of the top 15 genes important for the development of AEs is listed above in Table 2. These genes and their associated pathways may provide insight into the pathogenesis of AEs. In addition to the TLR pathway, Notch, NF-κB and IL-1 signaling were common themes. Four of the top 15 genes (*RBPJ*, *TLR2*, *ALDH1A2* and *APLP2*) are involved in TLR/Notch signaling. The Notch pathway is involved in development and is conserved from *Drosophila* to mammals. RBPJ is involved in the crosstalk between TLR and Notch signaling that is thought to help fine-tune the immune response through negative and/or positive feedback (S5 Fig) [49]. NF-κB is another downstream pathway of Notch signaling, and three of the top 15 genes (*PELI1*, *TLR2* and *LTBR*) are involved in NF-κB signaling. TLR2 is a receptor for the canonical NF-κB pathway, and PELI1 is involved in intracellular downstream signaling. The canonical pathway results in the release of pro-inflammatory cytokines, such as IL-6 and TNF-α. Thus, activation of this pathway is consistent with the cytokine profiles individuals who experienced moderate AEs after treatment. Interestingly, lymphotoxin beta receptor (LTBR) stimulation has been shown to enhance the LPS-induced expression of IL-8 via the combined action of NF-κB and IRF1 [50], and this is consistent with our results. Finally, IL-1 is clearly associated with the development of AEs. *IL-1RAP* was one of the top 15 genes identified by RF, and this gene is one of two co-receptors for IL-1. Additionally, expression of the second co-receptor *IL-1R1* was increased post-treatment in individuals with AEs. Both expression (this study) and plasma levels (our prior study [13]) of IL-1β increased after treatment in individuals with AEs. Inhibitors of the IL-1 pathway were also upregulated post-treatment in individuals with AEs. This included both increased expression and protein levels of *IL-1RA* and increased expression of the IL-1β decoy receptor *IL-1R2*. These results illustrate the importance of the balance between the pro-inflammatory effects of IL-1β and the anti-inflammatory effects of IL-1RA for AE development.

### Limitations of the study

One limitation of this study was that the RNA-seq was performed on mixed PBL samples. This makes it difficult to separate the effects of altered gene expression from the effects of changing cell type proportions. Separating different types of leukocytes was not feasible, because the samples were collected in rural Côte d’Ivoire and processed in a simple field lab. On the other hand, this study provides insight into the pathogenesis of post-treatment AEs. Additionally, it was possible to estimate the different cell subtypes present in PBLs using the RNA-seq data, so we could associate changes in gene expression with altered cell types to decrease the chance that the former was a directly result of the latter. For example, if the post-treatment AE transcriptional signature had been similar to a neutrophil gene expression profile it could have been caused by the increasing proportion of neutrophils post-treatment and not due to specific neutrophil activation during AEs. Another limitation was that we did not study an untreated control group, because it would have been unethical to withhold treatment from infected individuals. For the cytokine analysis we did not correct for multiple comparisons, however, the results are generally consistent with our previous findings [13], and this increases our confidence in the results. Based on the standard significance level of 0.05, approximately 3 differences would be expected to be significant by chance for 54 tests (27 cytokines measured pre- and post-treatment in two AE groups), whereas 17 comparisons were significant at the 0.05 level in this study. The semi-quantitative Filariasis Test Strip (FTS) was used to assay CFA in the field, whereas a quantitative ELISA was used to measure CFA levels in the laboratory setting in this study. Newer studies have demonstrated that tests that detect LF CFA (including FTS) can cross-react with *L*. *loa* antigens that circulate in blood from a subset of individuals with heavy infections [51], and with biological samples from animals infected with *L*. *loa* and *Onchocerca ochengi* [52, 53]. This cross-reactivity was not a concern for this study because *L*. *loa* is not endemic to Côte d’Ivoire, and the area of Côte d’Ivoire where the study was conducted is non-endemic for *O*. *volvulus*.

In future studies it would be interesting to measure *Wolbachia* DNA in pre and post-treatment samples from individuals with AEs after treatment of LF and onchocerciasis to try to correlate bacterial DNA release with host expression profiles post-treatment. *Wolbachia* DNA has been shown to increase post-treatment in individuals with AEs after treatment of LF [14]. Additionally, peak *Wolbachia* DNA levels have been shown to be correlated with AE reaction scores in individuals treated with DEC or IVM for onchocerciasis [54].

### Conclusions

This study included first global RNA-seq analysis of PBLs from LF-infected individuals, and it has provided novel insights into the pathogenesis of a clinically relevant problem. The samples were ideal for studying AEs after LF treatment, because each post-treatment sample was paired with a pre-treatment sample from the same individual. This internal control improved our ability to study the AE phenotype in humans. AEs represent a significant challenge for the global program to eliminate LF, and the fear of AEs in communities receiving MDA is a main factor that reduces compliance [25]. Minimizing the impact of AEs has therefore been identified as a key component for successful MDA programs [25]. More than 850 million individuals have been treated as part of GPELF, and a significant percentage of these individuals experience AEs.

This study has also provided a framework for investigating the host responses associated with severe AEs that occur after treatment of other filarial worms such as *O*. *volvulus* and *L*. *loa*. Treatment of other, more familiar infections can also result in severe AEs that are caused by host responses to dying pathogens. This Jarisch-Herxheimer reaction occurs after antibiotic treatment of spirochetal infections such as syphilis, Lyme disease, leptospirosis, and relapsing fever, and it is also hypothesized to be caused by the release of bacterial lipoproteins that activate TLR2 [55]. The transcriptomic response during the Jarisch-Herxheimer reaction has not been studied, so the dataset from this study could provide a valuable starting point for research on this related clinical problem.

To recap our major findings, this study has provided new insights regarding the pathogenesis of post-treatment AEs in LF-infected individuals. Our results are consistent with the hypothesis that a *Wolbachi*a lipoprotein triggers AEs by binding to TLR2-TLR6, but other uncharacterized filarial antigens might also play a role. Since TLR, NF-κB, and TNF pathways are involved, these pathways could potentially be targeted to prevent or treat AEs after LF treatment. We also found that high pre-treatment CFA levels were the best predictors of post-treatment AEs. This finding could be relevant for treatment-naïve areas with high LF infection prevalence and intensities. Individuals with high CFA levels pre-treatment (assessed with the FTS [56]) could be offered non-steroidal anti-inflammatory medications together with anti-filarial medications for home management of moderate or severe AEs. However, a positive FTS from an individual who resides in or has traveled to an area that is also endemic for *L*. *loa* needs to be interpreted with caution due to the issues of cross-reactivity mentioned above. Information from this study should allow program managers and drug distributors to reassure populations and communicate to them that AEs experienced after LF treatment are transient and caused by host responses to dying or injured parasites.

## Supporting information

S1 Fig Multiple filarial antigens increase post-treatment. Multiple filarial antigens with the AD12 carbohydrate epitope with different apparent molecular weights were present in post-treatment plasma from two persons, P1 and P2, whereas only the high molecular weight CFA (approximately 250 kDa) was present in pre-treatment plasma from the same individuals.
(DOCX)

S2 Fig Circulating immune complex levels. Mean circulating immune complex (CIC) levels ± standard error pre and post-treatment in individuals with no adverse events (AEs) (n = 33) and individuals with moderate AEs (n = 8). There was no significant difference between pre- and post-treatment values within the two AE groups (Wilcoxon signed-rank test), or between the two AE groups (Mann-Whitney U tests). AHG: aggregated human gamma globulin.
(DOCX)

S3 Fig Overview of TLR signaling. TLR signaling pathway with highlighted upregulated genes in black (based on DESeq2 analysis) and upregulated KEGG pathways (based on WebGestalt analysis) in red. Overrepresented transcription factors (based on i-CisTarget analysis) are in blue.
(DOCX)

S4 Fig Estimated leukocyte subtypes post-treatment. Percent change post-treatment of leukocyte subtypes in people with and without adverse events (AEs). T cells (CD4 naïve) decrease more and neutrophils increase more post-treatment in individuals with AEs (n = 9) compared to individuals with no AEs (n = 9). *P < 0.05, ** *P* < 0.01 by Mann-Whitney U tests.
(DOCX)

S5 Fig TLR and Notch. Crosstalk between the TLR signaling pathway and the Notch pathway. Expression and/or function of various components of the Notch pathways could be regulated by TLR signaling. Conversely, Notch pathway components positively or negatively modulate TLR-activated transcriptional, translational, and metabolic programs to finetune outcomes of immune responses Figure and text from Shang Y, Smith S, and Hu X. Role of Notch signaling in regulating innate immunity and inflammation in health and disease. *Protein Cell*. 2016;7(3):159–74
(DOCX)

S1 Table Metadata for all 95 individuals including which samples were used for all the experimental tests. (DOCX)

S2 Table Characteristics of each AE (adverse event) case and matched control with no AEs. (DOCX)

S3 Table Primer sequences. (DOCX)

S4 Table Age and sex distribution in the three adverse events (AEs) groups. ^A^Kruskal-Wallis H test, ^B^Chi-squared test.
(DOCX)

S5 Table Upregulated KEGG pathways post-treatment in individuals with adverse events (WebGestalt analysis). ^A^Kegg pathway name. C: the number of reference genes in the category. O: the number of genes in the gene set and also in the category. E: the expected number in the category. R: ratio of enrichment. *P*-value: *P*-value from hypergeometric test. FDR: *P*-value adjusted by the multiple test adjustment.
(DOCX)

S6 Table Enriched transcription factor binding sites in the 744 genes upregulated post-treatment in individuals with adverse events. (DOCX)

S7 Table Upregulated KEGG pathways pre-treatment in individuals that did not develop adverse events (Gene Set Enrichment Analysis). ^A^KEGG pathway. Size: Number of genes in the gene set after filtering out those genes not in the expression dataset. ES: enrichment score for the gene set; that is, the degree to which this gene set is overrepresented at the top or bottom of the ranked list of genes in the expression dataset. NES: normalized enrichment score; that is, the enrichment score for the gene set after it has been normalized across analyzed gene sets. NOM *P*-val: Nominal *P*-value; that is, the statistical significance of the enrichment score. The nominal *P*-value is not adjusted for gene set size or multiple hypothesis testing; therefore, it is of limited use in comparing gene sets. FDR q-val; false discovery rate; that is, the estimated probability that the normalized enrichment score represents a false positive finding. FWER *P*-val: Familywise-error rate; that is, a more conservatively estimated probability that the normalized enrichment score represents a false positive finding.
(DOCX)

S8 Table Variables in the random forest model (post-treatment fold change). (DOCX)

## References

[1] Simonsen PE, Fischer PU, Hoerauf A, Weil GJ. The Filariases In: Farrar J, Hotez PJ, Junghanss T, Kang G, Lalloo D, White NJ, editors. Manson's Tropical Diseases. 23rd ed: Elsevier; 2013 p. 737–65.

[2] WHO. Global programme to eliminate lymphatic filariasis: progress report, 2016. Global programme to eliminate lymphatic filariasis: progress report, 2016. 2017 2 October 2017; (No. 40, 2017, 91, 589–608):[20 p. p.]. Available from: https://www.who.int/lymphatic_filariasis/resources/who_wer9240/en/.

[3] Thomsen EK, Sanuku N, Baea M, Satofan S, Maki E, Lombore B, et al Efficacy, Safety, and Pharmacokinetics of Coadministered Diethylcarbamazine, Albendazole, and Ivermectin for Treatment of Bancroftian Filariasis. Clin Infect Dis. 2016;62(3):334–41. 10.1093/cid/civ882 26486704

[4] Ouattara AF KO, Bjerum C, Koudou BG, Meite A, Kazura JW, Weil G and King CL. High efficacy of singe dose of co-administered ivermecting, diethylcarbamazine and albendazole in treatment of lymphatic filariasis in Cote d'Ivoire. American Society of Tropical Medicine and Hygiene. 2016; 95(5):[600 p.]. Available from: https://www.astmh.org/ASTMH/media/Documents/ASTMH-2016-Annual-Meeting-Abstract-Book.pdf.

[5] Bjerum CM OA, Koudou BG, Meite A, Kazura JW, Weil G and King CL. The macrofilaricidal activity of a single dose of ivermectin, albendazole and diethylcarbamazine against Wuchereria bancrofti in Cote d'Ivoire. American Society of Tropical Medicine and Hygiene. 2016; 95(5):[599 p.]. Available from: https://www.astmh.org/ASTMH/media/Documents/ASTMH-2016-Annual-Meeting-Abstract-Book.pdf.

[6] Irvine MA, Stolk WA, Smith ME, Subramanian S, Singh BK, Weil GJ, et al Effectiveness of a triple-drug regimen for global elimination of lymphatic filariasis: a modelling study. Lancet Infect Dis. 2017;17(4):451–8. 10.1016/S1473-3099(16)30467-4 28012943

[7] King CL, Suamani J, Sanuku N, Cheng YC, Satofan S, Mancuso B, et al A Trial of a Triple-Drug Treatment for Lymphatic Filariasis. N Engl J Med. 2018;379(19):1801–10. 10.1056/NEJMoa1706854 30403937 PMC6194477

[8] WHO. Guideline—Alternative mass drug administration regimens to eliminate lymphatic filariasis. Lymphatic filariasis. November 2017:[xvii, 50 p. pp.]. Available from: https://www.who.int/lymphatic_filariasis/resources/9789241550161/en/. 29565523

[9] Horton J, Witt C, Ottesen EA, Lazdins JK, Addiss DG, Awadzi K, et al An analysis of the safety of the single dose, two drug regimens used in programmes to eliminate lymphatic filariasis. Parasitology. 2000;121 Suppl:S147–60. 11386686 10.1017/s0031182000007423

[10] Dreyer G, Pires ML, de Andrade LD, Lopes E, Medeiros Z, Tenorio J, et al Tolerance of diethylcarbamazine by microfilaraemic and amicrofilaraemic individuals in an endemic area of Bancroftian filariasis, Recife, Brazil. Trans R Soc Trop Med Hyg. 1994;88(2):232–6. 10.1016/0035-9203(94)90311-5 8036686

[11] Turner PF, Rockett KA, Ottesen EA, Francis H, Awadzi K, Clark IA. Interleukin-6 and tumor necrosis factor in the pathogenesis of adverse reactions after treatment of lymphatic filariasis and onchocerciasis. J Infect Dis. 1994;169(5):1071–5. 10.1093/infdis/169.5.1071 8169393

[12] Haarbrink M, Abadi GK, Buurman WA, Dentener MA, Terhell AJ, Yazdanbakhsh M. Strong association of interleukin-6 and lipopolysaccharide-binding protein with severity of adverse reactions after diethylcarbamazine treatment of microfilaremic patients. J Infect Dis. 2000;182(2):564–9. 10.1086/315735 10915090

[13] Andersen BJ, Kumar J, Curtis K, Sanuku N, Satofan S, King CL, et al Changes in Cytokine, Filarial Antigen, and DNA Levels Associated with Adverse Events Following Treatment of Lymphatic Filariasis. J Infect Dis. 2018;217(2):280–7. 10.1093/infdis/jix578 29149303 PMC5853815

[14] Cross HF, Haarbrink M, Egerton G, Yazdanbakhsh M, Taylor MJ. Severe reactions to filarial chemotherapy and release of Wolbachia endosymbionts into blood. Lancet. 2001;358(9296):1873–5. 10.1016/S0140-6736(01)06899-4 11741630

[15] Taylor MJ, Cross HF, Bilo K. Inflammatory responses induced by the filarial nematode Brugia malayi are mediated by lipopolysaccharide-like activity from endosymbiotic Wolbachia bacteria. J Exp Med. 2000;191(8):1429–36. 10.1084/jem.191.8.1429 10770808 PMC2193140

[16] Foster JM, Kumar S, Ganatra MB, Kamal IH, Ware J, Ingram J, et al Construction of bacterial artificial chromosome libraries from the parasitic nematode Brugia malayi and physical mapping of the genome of its Wolbachia endosymbiont. Int J Parasitol. 2004;34(6):733–46. 10.1016/j.ijpara.2004.02.001 15111095

[17] Foster J, Ganatra M, Kamal I, Ware J, Makarova K, Ivanova N, et al The Wolbachia genome of Brugia malayi: endosymbiont evolution within a human pathogenic nematode. PLoS Biol. 2005;3(4):e121 10.1371/journal.pbio.0030121 15780005 PMC1069646

[18] Turner JD, Langley RS, Johnston KL, Gentil K, Ford L, Wu B, et al Wolbachia lipoprotein stimulates innate and adaptive immunity through Toll-like receptors 2 and 6 to induce disease manifestations of filariasis. J Biol Chem. 2009;284(33):22364–78. 10.1074/jbc.M901528200 19458089 PMC2755959

[19] Tamarozzi F, Turner JD, Pionnier N, Midgley A, Guimaraes AF, Johnston KL, et al Wolbachia endosymbionts induce neutrophil extracellular trap formation in human onchocerciasis. Sci Rep. 2016;6:35559 10.1038/srep35559 27752109 PMC5067710

[20] Voronin D, Guimaraes AF, Molyneux GR, Johnston KL, Ford L, Taylor MJ. Wolbachia lipoproteins: abundance, localisation and serology of Wolbachia peptidoglycan associated lipoprotein and the Type IV Secretion System component, VirB6 from Brugia malayi and Aedes albopictus. Parasit Vectors. 2014;7:462 10.1186/s13071-014-0462-1 25287420 PMC4197220

[21] Stanilova SA, Slavov ES. Comparative study of circulating immune complexes quantity detection by three assays—CIF-ELISA, C1q-ELISA and anti-C3 ELISA. J Immunol Methods. 2001;253(1–2):13–21. 10.1016/s0022-1759(01)00370-2 11384665

[22] Zheng HJ, Tao ZH, Cheng WF, Wang SH, Cheng SH, Ye YM, et al Efficacy of ivermectin for control of microfilaremia recurring after treatment with diethylcarbamazine. II. Immunologic changes following treatment. Am J Trop Med Hyg. 1991;45(2):175–81. 1877712

[23] Ramaprasad P, Prasad GB, Harinath BC. Microfilaraemia, filarial antibody, antigen and immune complex levels in human filariasis before, during and after DEC therapy. A two-year follow-up. Acta Trop. 1988;45(3):245–55. 2903626

[24] Senbagavalli P, Anuradha R, Ramanathan VD, Kumaraswami V, Nutman TB, Babu S. Heightened measures of immune complex and complement function and immune complex-mediated granulocyte activation in human lymphatic filariasis. Am J Trop Med Hyg. 2011;85(1):89–96. 10.4269/ajtmh.2011.11-0086 21734131 PMC3122350

[25] Krentel A, Fischer PU, Weil GJ. A review of factors that influence individual compliance with mass drug administration for elimination of lymphatic filariasis. PLoS Negl Trop Dis. 2013;7(11):e2447 10.1371/journal.pntd.0002447 24278486 PMC3836848

[26] Weil GJ, Jain DC, Santhanam S, Malhotra A, Kumar H, Sethumadhavan KV, et al A monoclonal antibody-based enzyme immunoassay for detecting parasite antigenemia in bancroftian filariasis. J Infect Dis. 1987;156(2):350–5. 10.1093/infdis/156.2.350 3298458

[27] Kim D, Langmead B, Salzberg SL. HISAT: a fast spliced aligner with low memory requirements. Nat Methods. 2015;12(4):357–60. 10.1038/nmeth.3317 25751142 PMC4655817

[28] Liao Y, Smyth GK, Shi W. featureCounts: an efficient general purpose program for assigning sequence reads to genomic features. Bioinformatics. 2014;30(7):923–30. 10.1093/bioinformatics/btt656 24227677

[29] Love MI, Huber W, Anders S. Moderated estimation of fold change and dispersion for RNA-seq data with DESeq2. Genome Biol. 2014;15(12):550 10.1186/s13059-014-0550-8 25516281 PMC4302049

[30] Anderson MJ. A new method for non-parametric multivariate analysis of variance. Austral Ecology. 2001;26(1):32–46.

[31] Wang J, Vasaikar S, Shi Z, Greer M, Zhang B. WebGestalt 2017: a more comprehensive, powerful, flexible and interactive gene set enrichment analysis toolkit. Nucleic Acids Res. 2017;45(W1):W130–W7. 10.1093/nar/gkx356 28472511 PMC5570149

[32] Herrmann C, Van de Sande B, Potier D, Aerts S. i-cisTarget: an integrative genomics method for the prediction of regulatory features and cis-regulatory modules. Nucleic Acids Res. 2012;40(15):e114 10.1093/nar/gks543 22718975 PMC3424583

[33] Artyomov M. GeneQuery http://artyomovlab.wustl.edu/genequery/searcher/2017 [GeneQuery Website]. Available from: http://artyomovlab.wustl.edu/genequery/searcher/.

[34] Newman AM, Liu CL, Green MR, Gentles AJ, Feng W, Xu Y, et al Robust enumeration of cell subsets from tissue expression profiles. Nat Methods. 2015;12(5):453–7. 10.1038/nmeth.3337 25822800 PMC4739640

[35] Vandesompele J, De Preter K, Pattyn F, Poppe B, Van Roy N, De Paepe A, et al Accurate normalization of real-time quantitative RT-PCR data by geometric averaging of multiple internal control genes. Genome Biol. 2002;3(7):1–11. 10.1186/gb-2002-3-7-research0034 PMC126239 12184808

[36] Schmittgen TD, Livak KJ. Analyzing real-time PCR data by the comparative C(T) method. Nat Protoc. 2008;3(6):1101–8. 18546601 10.1038/nprot.2008.73

[37] Elkon R, Vesterman R, Amit N, Ulitsky I, Zohar I, Weisz M, et al SPIKE—a database, visualization and analysis tool of cellular signaling pathways. BMC Bioinformatics. 2008;9:110 10.1186/1471-2105-9-110 18289391 PMC2263022

[38] Ricklin D, Lambris JD. Complement in immune and inflammatory disorders: pathophysiological mechanisms. J Immunol. 2013;190(8):3831–8. 10.4049/jimmunol.1203487 23564577 PMC3623009

[39] Huang Y, Krein PM, Muruve DA, Winston BW. Complement factor B gene regulation: synergistic effects of TNF-alpha and IFN-gamma in macrophages. J Immunol. 2002;169(5):2627–35. 10.4049/jimmunol.169.5.2627 12193734

[40] Turner JD, Mand S, Debrah AY, Muehlfeld J, Pfarr K, McGarry HF, et al A randomized, double-blind clinical trial of a 3-week course of doxycycline plus albendazole and ivermectin for the treatment of Wuchereria bancrofti infection. Clin Infect Dis. 2006;42(8):1081–9. 10.1086/501351 16575724

[41] Hise AG, Daehnel K, Gillette-Ferguson I, Cho E, McGarry HF, Taylor MJ, et al Innate immune responses to endosymbiotic Wolbachia bacteria in Brugia malayi and Onchocerca volvulus are dependent on TLR2, TLR6, MyD88, and Mal, but not TLR4, TRIF, or TRAM. J Immunol. 2007;178(2):1068–76. 10.4049/jimmunol.178.2.1068 17202370

[42] Kim NJ, Ahn KB, Jeon JH, Yun CH, Finlay BB, Han SH. Lipoprotein in the cell wall of Staphylococcus aureus is a major inducer of nitric oxide production in murine macrophages. Mol Immunol. 2015;65(1):17–24. 10.1016/j.molimm.2014.12.016 25600878

[43] Schroder NW, Heine H, Alexander C, Manukyan M, Eckert J, Hamann L, et al Lipopolysaccharide binding protein binds to triacylated and diacylated lipopeptides and mediates innate immune responses. J Immunol. 2004;173(4):2683–91. 10.4049/jimmunol.173.4.2683 15294986

[44] Lee CC, Avalos AM, Ploegh HL. Accessory molecules for Toll-like receptors and their function. Nat Rev Immunol. 2012;12(3):168–79. 10.1038/nri3151 22301850 PMC3677579

[45] Campos MA, Almeida IC, Takeuchi O, Akira S, Valente EP, Procopio DO, et al Activation of Toll-like receptor-2 by glycosylphosphatidylinositol anchors from a protozoan parasite. J Immunol. 2001;167(1):416–23. 10.4049/jimmunol.167.1.416 11418678

[46] Becker I, Salaiza N, Aguirre M, Delgado J, Carrillo-Carrasco N, Kobeh LG, et al Leishmania lipophosphoglycan (LPG) activates NK cells through toll-like receptor-2. Mol Biochem Parasitol. 2003;130(2):65–74. 10.1016/s0166-6851(03)00160-9 12946842

[47] Hajishengallis G, Lambris JD. Crosstalk pathways between Toll-like receptors and the complement system. Trends Immunol. 2010;31(4):154–63. 10.1016/j.it.2010.01.002 20153254 PMC2849859

[48] Venugopal PG, Nutman TB, Semnani RT. Activation and regulation of toll-like receptors (TLRs) by helminth parasites. Immunol Res. 2009;43(1–3):252–63. 10.1007/s12026-008-8079-0 18982454 PMC3398210

[49] Shang Y, Smith S, Hu X. Role of Notch signaling in regulating innate immunity and inflammation in health and disease. Protein Cell. 2016;7(3):159–74. 10.1007/s13238-016-0250-0 26936847 PMC4791423

[50] Jang SW, Lim SG, Suk K, Lee WH. Activation of lymphotoxin-beta receptor enhances the LPS-induced expression of IL-8 through NF-kappaB and IRF-1. Immunol Lett. 2015;165(2):63–9. 10.1016/j.imlet.2015.04.001 25887375

[51] Pion SD, Montavon C, Chesnais CB, Kamgno J, Wanji S, Klion AD, et al Positivity of Antigen Tests Used for Diagnosis of Lymphatic Filariasis in Individuals Without Wuchereria bancrofti Infection But with High Loa loa Microfilaremia. Am J Trop Med Hyg. 2016;95(6):1417–23. 10.4269/ajtmh.16-0547 27729568 PMC5154460

[52] Pionnier NP, Sjoberg H, Chunda VC, Fombad FF, Chounna PW, Njouendou AJ, et al Mouse models of Loa loa. Nat Commun. 2019;10(1):1429 10.1038/s41467-019-09442-0 30926803 PMC6441053

[53] Wanji S, Amvongo-Adjia N, Njouendou AJ, Kengne-Ouafo JA, Ndongmo WP, Fombad FF, et al Further evidence of the cross-reactivity of the Binax NOW(R) Filariasis ICT cards to non-Wuchereria bancrofti filariae: experimental studies with Loa loa and Onchocerca ochengi. Parasit Vectors. 2016;9:267 10.1186/s13071-016-1556-8 27151313 PMC4858834

[54] Keiser PB, Reynolds SM, Awadzi K, Ottesen EA, Taylor MJ, Nutman TB. Bacterial endosymbionts of Onchocerca volvulus in the pathogenesis of posttreatment reactions. J Infect Dis. 2002;185(6):805–11. 10.1086/339344 11920298

[55] Butler T. The Jarisch-Herxheimer Reaction After Antibiotic Treatment of Spirochetal Infections: A Review of Recent Cases and Our Understanding of Pathogenesis. Am J Trop Med Hyg. 2017;96(1):46–52. 10.4269/ajtmh.16-0434 28077740 PMC5239707

[56] Chesnais CB, Vlaminck J, Kunyu-Shako B, Pion SD, Awaca-Uvon NP, Weil GJ, et al Measurement of Circulating Filarial Antigen Levels in Human Blood with a Point-of-Care Test Strip and a Portable Spectrodensitometer. Am J Trop Med Hyg. 2016;94(6):1324–9. 10.4269/ajtmh.15-0916 27114288 PMC4889752

