# Supplementary material for: Systems analysis-based assessment of post-treatment adverse events in lymphatic filariasis
Source: PLoS Negl Trop Dis. 2019 Sep 26;13(9):e0007697. doi: 10.1371/journal.pntd.0007697 (PMC6762072; doi:10.1371/journal.pntd.0007697)
Supplement: S1 Fig — Multiple filarial antigens with the AD12 carbohydrate epitope with different apparent molecular weights were present in post-treatment plasma from two persons, P1 and P2, whereas only the high molecular weight CFA (approximately 250 kDa) was present in pre-treatment plasma from the same individuals. (DOCX) [file pntd.0007697.s001.docx]

**S1 Fig. Multiple filarial antigens increase post-treatment**


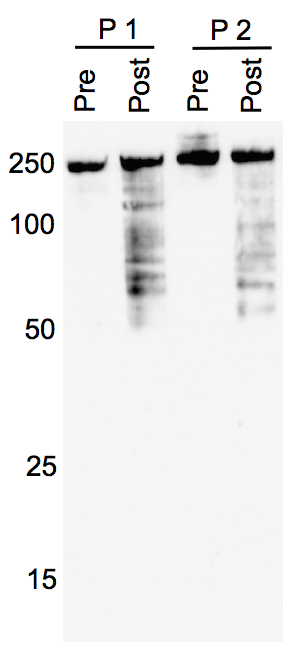


Multiple filarial antigens with the AD12 carbohydrate epitope with different apparent molecular weights were present in post-treatment plasma from two persons, P1 and P2, whereas only the high molecular weight CFA (approximately 250 kDa) was present in pre-treatment plasma from the same individuals.
