# Supplementary material for: Systems analysis-based assessment of post-treatment adverse events in lymphatic filariasis
Source: PLoS Negl Trop Dis. 2019 Sep 26;13(9):e0007697. doi: 10.1371/journal.pntd.0007697 (PMC6762072; doi:10.1371/journal.pntd.0007697)
Supplement: S2 Fig — Mean circulating immune complex (CIC) levels ± standard error pre and post-treatment in individuals with no adverse events (AEs) (n = 33) and individuals with moderate AEs (n = 8). There was no significant difference between pre- and post-treatment values within the two AE groups (Wilcoxon signed-rank test), or between the two AE groups (Mann-Whitney U tests). AHG: aggregated human gamma globulin. (DOCX) [file pntd.0007697.s002.docx]

**S2 Fig. Circulating immune complexes**

Mean circulating immune complex (CIC) levels ± standard error pre and post-treatment in individuals with no adverse events (AEs) (n=33) and individuals with moderate AEs (n=8). There was no significant difference between pre- and post-treatment values within the two AE groups (Wilcoxon signed-rank test), or between the two AE groups (Mann-Whitney U tests). AHG: aggregated human gamma globulin.
