# Supplementary material for: Systems analysis-based assessment of post-treatment adverse events in lymphatic filariasis
Source: PLoS Negl Trop Dis. 2019 Sep 26;13(9):e0007697. doi: 10.1371/journal.pntd.0007697 (PMC6762072; doi:10.1371/journal.pntd.0007697)
Supplement: S3 Fig — TLR signaling pathway with highlighted upregulated genes in black (based on DESeq2 analysis) and upregulated KEGG pathways (based on WebGestalt analysis) in red. Overrepresented transcription factors (based on i-CisTarget analysis) are in blue. (DOCX) [file pntd.0007697.s003.docx]

**S3 Fig. Overview of TLR signaling**

TLR signaling pathway with highlighted upregulated genes in black (based on DESeq2 analysis) and upregulated KEGG pathways (based on WebGestalt analysis) in red. Overrepresented transcription factors (based on i-CisTarget analysis) are in blue.
