# Supplementary material for: Systems analysis-based assessment of post-treatment adverse events in lymphatic filariasis
Source: PLoS Negl Trop Dis. 2019 Sep 26;13(9):e0007697. doi: 10.1371/journal.pntd.0007697 (PMC6762072; doi:10.1371/journal.pntd.0007697)
Supplement: S4 Fig — Percent change post-treatment of leukocyte subtypes in people with and without adverse events (AEs). T cells (CD4 naïve) decrease more and neutrophils increase more post-treatment in individuals with AEs (n = 9) compared to individuals with no AEs (n = 9). *P < 0.05, ** P < 0.01 by Mann-Whitney U tests. (DOCX) [file pntd.0007697.s004.docx]

**S4 Fig. Estimated leukocyte subtypes post-treatment.**

Median percent change post-treatment of leukocyte subtypes in people with and without adverse events (AEs). T cells (CD4 naïve) decrease more and neutrophils increase more post-treatment in individuals with moderate AEs (n=9) compared to individuals with no AEs (n=9). **P* < 0.05, ** *P* < 0.01 by Mann-Whitney U tests.
