# Supplementary material for: Systems analysis-based assessment of post-treatment adverse events in lymphatic filariasis
Source: PLoS Negl Trop Dis. 2019 Sep 26;13(9):e0007697. doi: 10.1371/journal.pntd.0007697 (PMC6762072; doi:10.1371/journal.pntd.0007697)
Supplement: S5 Fig — Crosstalk between the TLR signaling pathway and the Notch pathway. Expression and/or function of various components of the Notch pathways could be regulated by TLR signaling. Conversely, Notch pathway components positively or negatively modulate TLR-activated transcriptional, translational, and metabolic programs to finetune outcomes of immune responses Figure and text from Shang Y, Smith S, and Hu X. Role of Notch signaling in regulating innate immunity and inflammation in health and disease. Protein Cell. 2016;7(3):159–74 (DOCX) [file pntd.0007697.s005.docx]

**S5 Fig. TLR and Notch.**


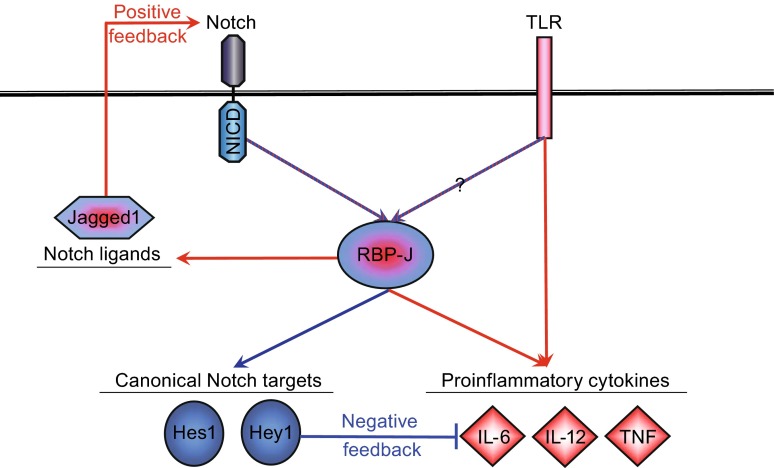


Crosstalk between the TLR signaling pathway and the Notch pathway. Expression and/or function of various components of the Notch pathways could be regulated by TLR signaling. Conversely, Notch pathway components positively or negatively modulate TLR-activated transcriptional, translational, and metabolic programs to finetune outcomes of immune responses

Figure and text from Shang Y, Smith S, and Hu X. Role of Notch signaling in regulating innate immunity and inflammation in health and disease. *Protein Cell*. 2016;7(3):159-74
