# Supplementary material for: Systems analysis-based assessment of post-treatment adverse events in lymphatic filariasis
Source: PLoS Negl Trop Dis. 2019 Sep 26;13(9):e0007697. doi: 10.1371/journal.pntd.0007697 (PMC6762072; doi:10.1371/journal.pntd.0007697)
Supplement: S1 Table — (DOCX) [file pntd.0007697.s006.docx]

**S1 Table. Metadata for all 95 individuals including which samples were used for each of the experimental tests.**

| **Metadata** | | | | | | **Qualitative/quantitative data collected per individual** | | | | | | |
| --- | --- | --- | --- | --- | --- | --- | --- | --- | --- | --- | --- | --- |
| Study ID | Treatment arm^A^ | Age | Sex | Mf/mL | AE^B^ group | CFA EIA | Western Blot | CIC elisa | C3/C4 and FB | LBP | Cytokines | RNA-seq |
| 422046 | 2 | 24 | m | 64 | 0 | yes | no | no | no | no | no | no |
| 422108 | 1 | 34 | m | 56 | 0 | yes | no | no | no | no | no | no |
| 412413 | 4 | 25 | m | 133.5 | 0 | yes | no | no | no | no | no | no |
| 422102 | 1 | 32 | m | 115 | 0 | yes | no | no | yes | yes | yes | yes |
| 422047 | 1 | 23 | m | 112 | 1 | yes | no | no | no | no | no | no |
| 422121 | 1 | 23 | m | 180 | 0 | yes | no | no | no | no | no | no |
| 422111 | 4 | 39 | m | 463.5 | 0 | yes | no | no | no | no | no | no |
| 412415 | 4 | 35 | m | 59.5 | 0 | yes | no | no | no | no | no | no |
| 422041 | 4 | 70 | f | 290 | 1 | yes | no | no | no | no | no | no |
| 422107 | 1 | 70 | m | 122 | 1 | yes | no | no | no | no | no | no |
| 412435 | 4 | 18 | m | 217 | 1 | yes | no | no | no | no | no | no |
| 422032 | 2 | 33 | m | 80 | 0 | yes | no | no | no | no | no | no |
| 412464 | 4 | 34 | m | 317 | 0 | yes | no | no | yes | yes | yes | yes |
| 412407 | 1 | 33 | m | 91.5 | 1 | yes | no | no | no | no | no | no |
| 10-4614 | 1 | 30 | m | 629 | 0 | yes | no | no | no | no | no | no |
| 10-4500 | 2 | 26 | m | 81 | 0 | yes | no | no | no | no | no | no |
| 422074 | 1 | 45 | m | 88 | 0 | yes | no | no | no | no | no | no |
| 422024 | 2 | 32 | m | 55 | 0 | yes | no | no | no | no | no | no |
| 412440 | 1 | 18 | m | 100.5 | 0 | yes | no | no | no | no | no | no |
| 412482 | 2 | 56 | m | 109 | 1 | yes | no | no | no | no | no | no |
| 422115 | 2 | 45 | m | 115.5 | 0 | yes | no | no | no | no | no | no |
| 412447 | 2 | 55 | m | 78.5 | 0 | yes | no | no | no | no | no | no |
| 422117 | 2 | 50 | f | 110 | 0 | yes | no | no | no | no | no | no |
| 422012 | 4 | 61 | m | 68 | 0 | yes | no | no | no | no | no | no |
| 422026 | 2 | 29 | m | 95 | 0 | yes | no | no | no | no | no | no |
| 412431 | 1 | 36 | m | 86.5 | 1 | yes | no | no | no | no | no | no |
| 412446 | 1 | 50 | f | 283.5 | 0 | yes | no | no | no | no | no | no |
| 412473 | 4 | 41 | m | 102.5 | 0 | yes | no | no | yes | yes | yes | yes |
| 422034 | 2 | 62 | m | 210 | 0 | yes | no | no | no | no | no | no |
| 412463 | 1 | 45 | f | 119.5 | 1 | yes | no | no | no | no | no | no |
| 422078 | 4 | 52 | m | 229 | 2 | yes | no | no | yes | yes | yes | yes |
| 412455 | 4 | 24 | m | 53.5 | 1 | yes | no | no | no | no | no | no |
| 422093 | 4 | 23 | m | 106.5 | 0 | yes | no | no | no | no | no | no |
| 422070 | 2 | 67 | f | 94 | 0 | yes | no | no | no | no | no | no |
| 412401 | 2 | 55 | m | 58.5 | 0 | yes | no | no | no | no | no | no |
| 10-4588 | 2 | 40 | m | 270.5 | 0 | yes | no | no | no | no | no | no |
| 422100 | 4 | 48 | m | 69.5 | 1 | yes | no | no | no | no | no | no |
| 412470 | 2 | 50 | m | 220 | 0 | yes | no | no | no | no | no | no |
| 422099 | 1 | 25 | m | 289.5 | 0 | yes | no | no | yes | yes | yes | yes |
| 412414 | 2 | 55 | m | 72.5 | 0 | yes | no | no | no | no | no | no |
| 422128 | 1 | 35 | m | 170.5 | 0 | yes | no | yes | no | no | no | no |
| 412434 | 2 | 65 | m | 703 | 0 | yes | no | yes | no | no | no | no |
| 422075 | 2 | 44 | m | 180.5 | 0 | yes | no | yes | yes | yes | yes | yes |
| 422112 | 4 | 35 | m | 274 | 1 | yes | no | no | no | no | no | no |
| 422079 | 2 | 50 | m | 968.5 | 0 | yes | no | yes | no | no | no | no |
| 422058 | 4 | 22 | m | 329 | 1 | yes | no | no | no | no | no | no |
| 412457 | 4 | 34 | m | 307.5 | 2 | yes | no | yes | yes | yes | yes | yes |
| 10-4651 | 1 | 36 | m | 106 | 0 | yes | no | yes | no | no | no | no |
| 422122 | 4 | 32 | m | 231.5 | 0 | yes | no | yes | no | no | no | no |
| 422125 | 4 | 64 | m | 79 | 0 | yes | no | yes | no | no | no | no |
| 412449 | 4 | 40 | f | 227 | 0 | yes | no | yes | no | no | no | no |
| 422028 | 4 | 65 | m | 70 | 0 | yes | no | yes | no | no | no | no |
| 422082 | 4 | 32 | m | 78.5 | 2 | yes | no | no | yes | yes | yes | yes |
| 412422 | 1 | 31 | m | 74 | 0 | yes | no | yes | no | no | no | no |
| 422083 | 1 | 62 | m | 611.5 | 0 | yes | no | yes | no | no | no | no |
| 422031 | 1 | 31 | m | 291 | 0 | yes | no | yes | no | no | no | no |
| 10-4698 | 2 | 32 | m | 518.5 | 0 | yes | no | yes | no | no | no | no |
| 422060 | 1 | 40 | m | 66 | 1 | yes | no | no | no | no | no | no |
| 422065 | 2 | 57 | m | 171 | 0 | yes | no | yes | no | no | no | no |
| 10-4536 | 1 | 19 | m | 151 | 0 | yes | no | yes | no | no | no | no |
| 422048 | 2 | 40 | m | 132 | 0 | yes | no | yes | no | no | no | no |
| 422144 | 2 | 39 | m | 575 | 0 | yes | no | yes | yes | yes | yes | yes |
| 412408 | 2 | 31 | f | 69.5 | 0 | yes | no | yes | no | no | no | no |
| 412467 | 1 | 52 | m | 240 | 0 | yes | no | yes | no | no | no | no |
| 412453 | 2 | 27 | m | 255 | 0 | yes | no | yes | no | no | no | no |
| 412423 | 2 | 25 | m | 221 | 1 | yes | no | no | no | no | no | no |
| 422080 | 1 | 28 | m | 131 | 1 | yes | no | no | no | no | no | no |
| 422126 | 2 | 32 | m | 444.5 | 0 | yes | no | yes | no | no | no | no |
| 412428 | 1 | 31 | m | 292.5 | 1 | yes | no | no | no | no | no | no |
| 412469 | 4 | 18 | f | 265 | 1 | yes | no | no | no | no | no | no |
| 422091 | 1 | 35 | m | 384 | 1 | yes | no | no | yes | yes | yes | yes |
| 412441 | 1 | 24 | m | 450 | 0 | yes | no | yes | no | no | no | no |
| 10-4681 | 4 | 35 | m | 165 | 1 | yes | no | no | no | no | no | no |
| 422094 | 1 | 26 | m | 423.5 | 0 | yes | no | yes | no | no | no | no |
| 412442 | 2 | 46 | f | 151.5 | 0 | yes | no | yes | no | no | no | no |
| 412426 | 2 | 18 | f | 307 | 0 | yes | no | yes | no | no | no | no |
| 422076 | 1 | 35 | m | 348.5 | 2 | yes | yes | yes | yes | yes | yes | yes |
| 412476 | 1 | 35 | m | 261 | 1 | yes | no | no | no | no | no | no |
| 412468 | 1 | 23 | f | 466.5 | 0 | yes | no | yes | yes | yes | yes | yes |
| 08-2665 | 4 | 32 | m | 222 | 1 | yes | no | no | no | no | no | no |
| 412458 | 2 | 24 | m | 230 | 1 | yes | no | no | no | no | no | no |
| 412411 | 1 | 29 | m | 560.5 | 2 | yes | yes | yes | yes | yes | yes | yes |
| 06-4282 | 4 | 39 | m | 1498 | 0 | yes | no | yes | no | no | no | no |
| 412485 | 4 | 47 | m | 590 | 0 | yes | no | yes | no | no | no | no |
| 412462 | 4 | 33 | m | 246 | 0 | yes | no | yes | no | no | no | no |
| 422030 | 4 | 20 | m | 101 | 0 | yes | no | yes | no | no | no | no |
| 422036 | 4 | 63 | m | 142 | 1 | yes | no | no | no | no | no | no |
| 422098 | 1 | 30 | m | 144.5 | 0 | yes | no | yes | yes | yes | yes | yes |
| 422134 | 1 | 60 | m | 700 | 2 | yes | yes | yes | yes | yes | yes | yes |
| 422087 | 2 | 35 | m | 860 | 0 | yes | yes | yes | no | no | no | no |
| 412412 | 4 | 69 | m | 503 | 2 | yes | yes | yes | yes | yes | yes | yes |
| 422089 | 4 | 30 | f | 263.5 | 2 | yes | yes | yes | yes | yes | yes | yes |
| 412452 | 2 | 25 | m | 944 | 0 | yes | yes | yes | no | no | no | no |
| 422116 | 4 | 51 | m | 328.5 | 1 | yes | yes | no | no | no | no | no |
| 422077 | 4 | 48 | m | 659.5 | 2 | yes | yes | yes | yes | yes | yes | yes |

^A^Treatment arm: 1=ALB/IVM, 2=ALB, 4=ALB/IVM/DEC

^B^AE group: 0=no AEs, 1=mild AEs, 2=moderate AEs
