# Supplementary material for: Systems analysis-based assessment of post-treatment adverse events in lymphatic filariasis
Source: PLoS Negl Trop Dis. 2019 Sep 26;13(9):e0007697. doi: 10.1371/journal.pntd.0007697 (PMC6762072; doi:10.1371/journal.pntd.0007697)
Supplement: S2 Table — (DOCX) [file pntd.0007697.s007.docx]

**S2 Table. Characteristics of each moderate AE (adverse event) case and matched control with no AEs**

| ID of AE case | Characteristics | With AEs | No AEs |
| --- | --- | --- | --- |
| 5 | Sex  Age  Mf/mL  Treatment | Male  35  349  IVM/ALB | Male  30  145  IVM/ALB |
| 6 | Sex  Age  Mf/mL  Treatment | Male  60  700  IVM/ALB | Male  32  115  IVM/ALB |
| 7 | Sex  Age  Mf/mL  Treatment | Female  32  264  IVM/DEC/ALB | Female  23  467  IVM/ALB |
| 8 | Sex  Age  Mf/mL  Treatment | Male  48  660  IVM/DEC/ALB | Male  39  575  ALB |
| 9 | Sex  Age  Mf/mL  Treatment | Male  53  229  IVM/DEC/ALB | Male  41  103  IVM/DEC/ALB |
| 10 | Sex  Age  Mf/mL  Treatment | Male  34  308  IVM/DEC/ALB | Male  34  317  IVM/DEC/ALB |
| 11 | Sex  Age  Mf/mL  Treatment | Male  29  560  IVM/ALB | Male  35  384  IVM/ALB |
| 12 | Sex  Age  Mf/mL  Treatment | Male  69  503  IVM/DEC/ALB | Male  44  181  ALB |
| 17 | Sex  Age  Mf/mL  Treatment | Male  32  79  IVM/DEC/ALB | Male  25  181  ALB |
