# Supplementary material for: Systems analysis-based assessment of post-treatment adverse events in lymphatic filariasis
Source: PLoS Negl Trop Dis. 2019 Sep 26;13(9):e0007697. doi: 10.1371/journal.pntd.0007697 (PMC6762072; doi:10.1371/journal.pntd.0007697)
Supplement: S3 Table — (DOCX) [file pntd.0007697.s008.docx]

**S3 Table. Primer sequences**

| Gene | Forward Primer Sequence | Reverse Primer Sequence | Company | KicqStart Primer Pair ID |
| --- | --- | --- | --- | --- |
| YWAZ | 5'- ACT TTT GGT ACA TTG TGG CTT CAA -3' | 5'- CCG CCA GGA CAA ACC AGT AT -3' | IDT | NA |
| SDHA | 5'- TGG GAA CAA GAG GGC ATC TG -3' | 5'- CCA CCA CTG CAT CAA ATT CAT G -3' | IDT | NA |
| ACTB | 5'- CTC TGG CTC CTA GCA CCA TGA AGA -3' | 5'- GTA AAA CGC AGC TCA GTA ACA GTC CG -3' | IDT | NA |
| HPRT1 | 5'- TGC AGA CTT TGC TTT CCT TGG TCA GG -3' | 5'- CCA ACA CTT CGT GGG GTC CTT TTC A -3' | IDT | NA |
| DIP2B | 5'- AGT CCT TCT CTA AGC TCT TC -3' | 5'- GGT TCC CTG TAA ACA TAT TGC -3' | Sigma | H_DIP2B_1 |
| ZCCHC6 | 5'- AAG CTT TGC AGT ATA GAT CG -3' | 5'- TAG ATA TAC AGG CAA AAG GGG -3' | Sigma | H_ZCCHC6_1 |
| RBPJ | 5'- AAT TCA ATT TCA GGC CAC TC -3' | 5'- CTA ATG ATT GTC CAG GAA GC -3' | Sigma | H_RBPJ_1 |
| PELI1 | 5'- AGG CAA TAA GCA ACA AAG AC -3' | 5'- GTC ATG AGT ATA TTC AAC CAC C -3' | Sigma | H_PELI1_1 |
| FNDC3B | 5'- CAC AGG TTT CTA ATA TTC AGG C -3' | 5'- CTT GTA TTT TCC ATC TCG TCC -3' | Sigma | H_FNDC3B_1 |
| TLR2 | 5'- CTT TCA ACT GGT AGT TGT GG -3' | 5'- GGA ATG GAG TTT AAA GAT CCT G -3' | Sigma | H_TLR2_1 |
| LTBR | 5'- CAT TTC TGG AGA TGT TTC CC -3' | 5'- TAG ATG TTG CCA GTG ATA GTC -3' | Sigma | H_LTBR_1 |
| NT5C2 | 5'- CAG CGA GAT GAT ACT GAA AG -3' | 5'- ACA ACT GGT ATA TCT GGG AC -3' | Sigma | H_NT5C2_1 |
