# Supplementary material for: Systems analysis-based assessment of post-treatment adverse events in lymphatic filariasis
Source: PLoS Negl Trop Dis. 2019 Sep 26;13(9):e0007697. doi: 10.1371/journal.pntd.0007697 (PMC6762072; doi:10.1371/journal.pntd.0007697)
Supplement: S4 Table — AKruskal-Wallis H test, BChi-squared test. (DOCX) [file pntd.0007697.s009.docx]

**S4 Table. Age and sex distribution in the three adverse events (AEs) groups**

|  | No AEs (n=62) | Mild AEs (n=24) | Moderate AEs (n=9) | *P*-value |
| --- | --- | --- | --- | --- |
| Age | 38.5 | 37.4 | 43.2 | 0.58^A^ |
| Sex (male) | 87.1% | 87.5% | 88.8% | 0.99^B^ |

^A^Kruskal-Wallis H test

^B^Chi-squared test
