# Supplementary material for: Systems analysis-based assessment of post-treatment adverse events in lymphatic filariasis
Source: PLoS Negl Trop Dis. 2019 Sep 26;13(9):e0007697. doi: 10.1371/journal.pntd.0007697 (PMC6762072; doi:10.1371/journal.pntd.0007697)
Supplement: S5 Table — AKegg pathway name. C: the number of reference genes in the category. O: the number of genes in the gene set and also in the category. E: the expected number in the category. R: ratio of enrichment. P-value: P-value from hypergeometric test. FDR: P-value adjusted by the multiple test adjustment. (DOCX) [file pntd.0007697.s010.docx]

**S5 Table. Upregulated KEGG pathways post-treatment in individuals with moderate adverse events (WebGestalt analysis).**

| **Geneset** | **Description^A^** | **C** | **O** | **E** | **R** | ***P*-Value** | **FDR** |
| --- | --- | --- | --- | --- | --- | --- | --- |
| hsa04380 | Osteoclast differentiation | 127 | 34 | 6.918 | 4.915 | 2.11E-15 | 6.39E-13 |
| hsa05152 | Tuberculosis | 167 | 33 | 9.097 | 3.628 | 5.25E-11 | 7.96E-09 |
| hsa05164 | Influenza A | 170 | 29 | 9.260 | 3.132 | 2.78E-08 | 2.81E-06 |
| hsa04666 | Fc gamma R-mediated phagocytosis | 91 | 20 | 4.957 | 4.035 | 5.83E-08 | 4.33E-06 |
| hsa04621 | NOD-like receptor signaling pathway | 167 | 28 | 9.097 | 3.078 | 7.15E-08 | 4.33E-06 |
| hsa05162 | Measles | 133 | 24 | 7.245 | 3.313 | 1.53E-07 | 7.74E-06 |
| hsa04145 | Phagosome | 147 | 25 | 8.007 | 3.122 | 2.77E-07 | 1.15E-05 |
| hsa05140 | Leishmaniasis | 66 | 16 | 3.595 | 4.451 | 3.03E-07 | 1.15E-05 |
| hsa05134 | Legionellosis | 54 | 14 | 2.941 | 4.760 | 6.98E-07 | 2.35E-05 |
| hsa04064 | NF-kappa B signaling pathway | 89 | 18 | 4.848 | 3.713 | 1.01E-06 | 3.06E-05 |
| hsa05120 | Epithelial cell signaling in Helicobacter pylori infection | 67 | 14 | 3.649 | 3.836 | 1.10E-05 | 0.00030 |
| hsa04062 | Chemokine signaling pathway | 181 | 25 | 9.859 | 2.536 | 1.38E-05 | 0.00035 |
| hsa05132 | Salmonella infection | 82 | 15 | 4.467 | 3.358 | 2.90E-05 | 0.00068 |
| hsa04650 | Natural killer cell mediated cytotoxicity | 123 | 19 | 6.700 | 2.836 | 3.18E-05 | 0.00069 |
| hsa05321 | Inflammatory bowel disease (IBD) | 62 | 12 | 3.377 | 3.553 | 0.00010 | 0.00209 |
| hsa05168 | Herpes simplex infection | 177 | 22 | 9.641 | 2.282 | 0.00022 | 0.00423 |
| hsa04060 | Cytokine-cytokine receptor interaction | 258 | 28 | 14.053 | 1.992 | 0.00034 | 0.00602 |
| hsa04640 | Hematopoietic cell lineage | 92 | 14 | 5.011 | 2.794 | 0.00041 | 0.00687 |
| hsa05130 | Pathogenic Escherichia coli infection | 55 | 10 | 2.996 | 3.338 | 0.00066 | 0.01049 |
| hsa04668 | TNF signaling pathway | 108 | 15 | 5.883 | 2.550 | 0.00070 | 0.01055 |
| hsa04630 | Jak-STAT signaling pathway | 156 | 19 | 8.497 | 2.236 | 0.00077 | 0.01104 |
| hsa05145 | Toxoplasmosis | 110 | 15 | 5.992 | 2.503 | 0.00085 | 0.01166 |
| hsa04664 | Fc epsilon RI signaling pathway | 69 | 11 | 3.758 | 2.927 | 0.00113 | 0.01492 |
| hsa04662 | B cell receptor signaling pathway | 70 | 11 | 3.813 | 2.885 | 0.00128 | 0.01590 |
| hsa05150 | Staphylococcus aureus infection | 50 | 9 | 2.724 | 3.305 | 0.00131 | 0.01590 |
| hsa05133 | Pertussis | 72 | 11 | 3.922 | 2.805 | 0.00162 | 0.01889 |
| hsa05146 | Amoebiasis | 95 | 13 | 5.175 | 2.512 | 0.00179 | 0.02010 |
| hsa05160 | Hepatitis C | 131 | 16 | 7.136 | 2.242 | 0.00190 | 0.02059 |
| hsa05161 | Hepatitis B | 145 | 17 | 7.898 | 2.152 | 0.00217 | 0.02270 |
| hsa04010 | MAPK signaling pathway | 252 | 25 | 13.726 | 1.821 | 0.00250 | 0.02528 |
| hsa04620 | Toll-like receptor signaling pathway | 101 | 13 | 5.501 | 2.363 | 0.00312 | 0.03048 |
| hsa04210 | Apoptosis | 138 | 16 | 7.517 | 2.129 | 0.00325 | 0.03079 |
| hsa04066 | HIF-1 signaling pathway | 102 | 13 | 5.556 | 2.340 | 0.00340 | 0.03124 |
| hsa04670 | Leukocyte transendothelial migratio | 116 | 14 | 6.319 | 2.216 | 0.00400 | 0.03565 |
| hsa04071 | Sphingolipid signaling pathway | 118 | 14 | 6.427 | 2.178 | 0.00467 | 0.04045 |

^A^Kegg pathway name. C: the number of reference genes in the category. O: the number of genes in the gene set and also in the category. E: the expected number in the category. R: ratio of enrichment. *P*-value: *P*-value from hypergeometric test. FDR: *P*-value adjusted by the multiple test adjustment.
