# Supplementary material for: Systems analysis-based assessment of post-treatment adverse events in lymphatic filariasis
Source: PLoS Negl Trop Dis. 2019 Sep 26;13(9):e0007697. doi: 10.1371/journal.pntd.0007697 (PMC6762072; doi:10.1371/journal.pntd.0007697)
Supplement: S6 Table — (DOCX) [file pntd.0007697.s011.docx]

**S6 Table. Enriched transcription factor binding sites in the 744 genes upregulated post-treatment in individuals with moderate adverse events.**

| Transcription factor binding site | Normalized enrichment score |
| --- | --- |
| *STAT1* | 7.74 |
| *STAT2* | 7.23 |
| *SPI1* | 6.40 |
| *STAT3* | 4.43 |
| *IRF1* | 4.24 |
| *CEBPB* | 3.50 |
