# Supplementary material for: Systems analysis-based assessment of post-treatment adverse events in lymphatic filariasis
Source: PLoS Negl Trop Dis. 2019 Sep 26;13(9):e0007697. doi: 10.1371/journal.pntd.0007697 (PMC6762072; doi:10.1371/journal.pntd.0007697)
Supplement: S7 Table — AKEGG pathway. Size: Number of genes in the gene set after filtering out those genes not in the expression dataset. ES: enrichment score for the gene set; that is, the degree to which this gene set is overrepresented at the top or bottom of the ranked list of genes in the expression dataset. NES: normalized enrichment score; that is, the enrichment score for the gene set after it has been normalized across analyzed gene sets. NOM P-val: Nominal P-value; that is, the statistical significance of the enrichment score. The nominal P-value is not adjusted for gene set size or multiple hypothesis testing; therefore, it is of limited use in comparing gene sets. FDR q-val; false discovery rate; that is, the estimated probability that the normalized enrichment score represents a false positive finding. FWER P-val: Familywise-error rate; that is, a more conservatively estimated probability that the normalized enrichment score represents a false positive finding. (DOCX) [file pntd.0007697.s012.docx]

**S7 Table. Published expression profiles similar to the transcriptional signature of post-treatment adverse events (GeneQuery results).**

| **#** | **Experiment title** | **Module** | **log10**  **(adj.pvalue)** | **Overlap** | **GSE^A^** |
| --- | --- | --- | --- | --- | --- |
| 1 | The novel antisense Bcl-2 inhibitor SPC2996 causes rapid leukemic cell clearance and immune activation in chronic lymphocytic leukemia | 1 | -274.49 | 481/1288 | GSE27858 |
| 2 | Expression data from normal and Malignant hematopoietic cells | 2 | -257.07 | 507/1451 | GSE48558 |
| 3 | Expression data from whole blood samples of Rwandan adults with mild malaria with matched sample thirty days later (convalescence) | 4 | -218.31 | 366/726 | GSE64338 |
| 4 | Blood Transcriptional Profiles in Active and Latent Tuberculosis UK (Training Set) | 1 | -212.29 | 373/820 | GSE19439 |
| 5 | Distinct, non-overlapping gene panels of peripheral blood gene expression predict response to infliximab therapy in rheumatoid arthritis and Crohn's disease | 2 | -206.75 | 407/983 | GSE42296 |
| 6 | Genome-wide analysis of gene expression profiles in individuals infected with the Human T-Lymphotropic virus Type 1 (HTLV-1) | 2 | -206.66 | 335/723 | GSE29333 |
| 7 | Genomic profiles for human peripheral blood T cells, B cells, natural killer cells, monocytes, and polymorphonuclear cells: comparisons to ischemic stroke, migraine, and Tourette syndrome | 2 | -206.44 | 470/1398 | GSE72642 |
| 8 | Genome-wide analysis of whole blood transcriptional response to community-acquired Staphylococcus aureus infection in vivo | 2 | -205.49 | 411/1111 | GSE30119 |
| 9 | Expression data from obese children (boys) | 2 | -203.16 | 420/1182 | GSE41505 |
| 10 | Application of genome-wide expression analysis to human health &amp; disease | 2 | -201.5 | 383/977 | GSE2328 |
| 11 | Trivalent Inactivated Influenza Vaccine (TIV) and Live Attenuated Influenza Vaccine (LAIV) Induce Different B cell and Transcriptional Responses in Children | 1 | -195.47 | 382/1041 | GSE52005 |
| 12 | Upregulation of immunoproteasome subunits in myositis indicates active inflammation with involvement of antigen presenting cells, CD8 T-cells and IFN | 1 | -193.91 | 505/1629 | GSE58173 |
| 13 | An Expression Atlas of Human Primary Cells: Inference of Gene Function from Coexpression Networks | 2 | -193.1 | 388/968 | GSE49910 |
| 14 | Genome wide transcriptional profiling of HIV positive and negative children with active tuberculosis, latent TB infection and other diseases from Kenya | 1 | -189.4 | 354/836 | GSE39939 |
| 15 | Transcriptome from circulating cells suggests dysregulated pathways associated with long-term recurrent events following first-time myocardial infarction. | 3 | -188.33 | 297/492 | GSE48060 |
| 16 | Whole Blood Transcriptional Response to Pediatric Influenza Infection | 1 | -186.67 | 429/1446 | GSE29366 |
| 17 | The genomic architecture of host whole blood transcriptional response to malaria infection | 1 | -183.73 | 355/876 | GSE34404 |
| 18 | Activation of the Interferon Pathway is Dependent upon Autoantibodies in African-American SLE Patients, but not in European-American SLE Patients | 2 | -180.86 | 328/645 | GSE50635 |
| 19 | Transcriptional analysis of whole blood, primary fibroblasts, and PBMCs upon TNF-alpha or IL-1beta stimulation from HOIL-1-deficient patients | 2 | -179.93 | 301/630 | GSE40752 |
| 20 | Transcriptional analysis of whole blood in patients with auto-inflammatory disorders | 2 | -179.93 | 301/630 | GSE40561 |
| 21 | Gene expression profiles in white blood cell subgroups | 2 | -178.48 | 368/1022 | GSE4251 |
| 22 | Whole blood transcriptional signature distinguishes viral infection from bacterial infection in febrile young children. | 1 | -169.83 | 401/1287 | GSE40396 |
| 23 | Combination of peripheral blood gene expression profiles and clinical parameters predicts response for tocilizumab (anti-IL6) treatment in rheumatoid arthritis | 2 | -169.23 | 433/1354 | GSE25160 |
| 24 | Gene expression in RNP autoantibody+ systemic lupus erythematosus (SLE) patient blood | 2 | -167.57 | 466/1457 | GSE61635 |
| 25 | Leukocytes Gene Expression in Correlation to Plasma Lipid Levels | 2 | -166.2 | 468/1540 | GSE3059 |
| 26 | Whole blood mRNA expression profiling of host molecular networks in neonatal sepsis | 3 | -162.93 | 318/773 | GSE25504 |
| 27 | Peripheral blood RNA gene expression profiling in patients with bacterial meningitis | 1 | -161.12 | 561/2264 | GSE40586 |
| 28 | Olfactomedin 4 serves as a marker for disease severity in pediatric Respiratory Syncytial Virus (RSV) infection | 1 | -160.82 | 360/1005 | GSE69606 |
| 29 | Gene expression analysis of urine sediment: evaluation for potential noninvasive markers of interstitial cystitis/bladder pain syndrome | 2 | -160.66 | 442/1482 | GSE28242 |
| 30 | Molecular phenotype of G-CSF- or pegylated G-CSF-mobilized CD34+ cells | 1 | -158.71 | 335/1338 | GSE4688 |
| 31 | Lipid metabolism genes in contralateral unaffected breast and estrogen receptor status of breast cancer | 3 | -158.25 | 275/653 | GSE41400 |
| 32 | Transcriptional Profiling is Superior to Procalcitonin to Discriminate Bacterial vs. Viral Lower Respiratory Tract Infections in Hospitalized Adults | 2 | -155.06 | 410/1285 | GSE60244 |
| 33 | Genome-wide gene expression analysis of the whole blood leukocyte transcriptional response to endotoxin treatment | 2 | -153.52 | 465/1957 | GSE36177 |
| 34 | Integrative DNA methylation and gene expression analyses identify DNA packaging and epigenetic regulatory genes associated with low motility sperm | 2 | -153.51 | 382/1521 | GSE26982 |
| 35 | Expression data from peripheral blood from healthy and predisposed individuals | 3 | -153.16 | 316/737 | GSE6351 |
| 36 | Gene expression profiling in pediatric meningococcal sepsis reveals dynamic changes in NK-cell and cytotoxic molecules | 2 | -151.64 | 472/1700 | GSE11755 |
| 37 | Gene expression in human myeloid cells | 2 | -150.4 | 294/870 | GSE12803 |
| 38 | Blood Transcriptional Profiles of Active TB (UK Test Set Separated) | 1 | -148.3 | 420/1546 | GSE19443 |
| 39 | Abnormal Expression Changes in AML | 2 | -147.35 | 372/1360 | GSE9476 |
| 40 | Whole Genome Expression Array in Human T-cell Acute Lymphoblastic Leukemia | 3 | -146.13 | 306/964 | GSE46170 |
| 41 | Expression in induced sputum during acute exacerbations in asthmatic children with/without chronic airflow obstruction | 3 | -145.78 | 394/1304 | GSE19903 |
| 42 | Nocturnal Hemodialysis Improves Erythropoietin Responsiveness | 1 | -145.67 | 435/1466 | GSE11227 |
| 43 | Allele-specific FKBP5 DNA demethylation: a molecular mediator of gene-childhood trauma interactions | 4 | -144.67 | 244/473 | GSE42002 |
| 44 | A network-based analysis of systemic inflammation in humans | 2 | -143.24 | 428/1323 | GSE3284 |
| 45 | Interferon Signature in the Blood in Inflammatory Common Variable Immune Deficiency | 2 | -140.97 | 289/691 | GSE51406 |
| 46 | Interferon Signature in the Blood in Inflammatory Common Variable Immune Deficiency [Test Set] | 2 | -140.97 | 289/691 | GSE51404 |
| 47 | Changes in the transcriptome of circulating neutrophils and skeletal muscle in response to endurance exercise | 4 | -139.78 | 264/636 | GSE43856 |
| 48 | Human immune cell transcriptome | 1 | -138.97 | 268/648 | GSE3982 |
| 49 | Aberrant expressed genes in AML | 1 | -138.74 | 413/1863 | GSE37307 |
| 50 | Genome-wide profiling of DNA methylation and expression identifies CIMP in myelodysplastic syndrome | 1 | -137.95 | 384/1487 | GSE51759 |
| 51 | Genome-wide profiling of DNA methylation and expression identifies CIMP in myelodysplastic syndrome [Agilent] | 1 | -137.95 | 384/1487 | GSE51757 |
| 52 | Identification of a B cell signature associated with renal transplant Tolerance in humans | 3 | -136.29 | 258/491 | GSE22229 |
| 53 | Whole Blood Gene Expression Profiling Predicts Therapeutic Response in Polyarticular Juvenile Idiopathic Arthritis at 4 Months | 2 | -134.67 | 201/330 | GSE55319 |
| 54 | Gene expression annalysis of peripheral blood cells in patients with chronic kidney disease | 3 | -134.15 | 283/622 | GSE70528 |
| 55 | Genome-wide gene expression profiling of human narcolepsy | 3 | -132.49 | 257/617 | GSE21592 |
| 56 | The Plasma Cell Signature in Autoimmune Disease | 4 | -131.22 | 203/306 | GSE45537 |
| 57 | A Predictive Response Signature to Infliximab Treatment in Ulcerative Colitis | 2 | -129.53 | 215/415 | GSE12251 |
| 58 | Atherosclerosis study using female peripheral blood samples | 3 | -129.46 | 287/770 | GSE20129 |
| 59 | Transcriptome profiling of deciduas from pre-eclamptic and normotensive pregnancies | 2 | -129.38 | 375/1550 | GSE60438 |
| 60 | Expression data from multiple sclerosis patients in remission and relapse | 1 | -129.1 | 322/820 | GSE41890 |
| 61 | Peripheral blood gene expression changes during allergen inhalation challenge in atopic asthmatic individuals | 1 | -128.88 | 382/1257 | GSE24745 |
| 62 | Genome-wide analysis of gene expression profiles in individuals infected with the Human T-Lymphotropic virus Type 1 (HTLV-1) | 2 | -128.46 | 268/679 | GSE29333 |
| 63 | Cell Specific Expression &amp; Pathway Analyses Reveal Novel Alterations in Trauma-Related Human T-Cell &amp; Monocyte Pathways | 5 | -127.76 | 213/397 | GSE5580 |
| 64 | Age gene expression in Healthy leukocytes | 3 | -127.57 | 269/579 | GSE69832 |
| 65 | The transcriptional modulator H2AFY marks Huntington's disease activity in men and mice | 3 | -127.11 | 228/500 | GSE24250 |
| 66 | Reliability and stability of individual differences in gene expression | 3 | -126.8 | 265/590 | GSE14844 |
| 67 | Suppression of T Cell Activation and Collagen Accumulation by an Anti-IFNAR1 mAb, Anifrolumab, in Adult Patients with Systemic Sclerosis | 1 | -126.2 | 479/2146 | GSE65336 |
| 68 | Expression data from Fine Needle Aspiration (FNA) biopsies from breast cancer patients | 4 | -124.15 | 184/359 | GSE22597 |
| 69 | Effects of acute dietary zinc depletion on the gene expression profile of whole blood in human male adults | 3 | -124.14 | 214/453 | GSE33174 |
| 70 | Identification of time-dependent biomarkers and effects of exposure to volatile organic compounds using high-throughput analysis | 2 | -123.51 | 311/984 | GSE68909 |
| 71 | T cell responses to H1N1v and a longitudinal study of seasonal influenza vaccination | 3 | -123.24 | 246/560 | GSE58970 |
| 72 | Sarcoidosis-specific markers from whole blood gene expression | 2 | -122.42 | 297/742 | GSE19314 |
| 73 | Expression data from whole blood | 2 | -119.84 | 238/498 | GSE4488 |
| 74 | Gene expression data from 131 human subjects in Detroit, Michigan | 4 | -119.45 | 206/358 | GSE35571 |
| 75 | T cell responses to H1N1v and a longitudinal study of seasonal influenza vaccination - 2009 | 2 | -119.11 | 280/774 | GSE58943 |
| 76 | Whole blood mRNA expression profiling of host molecular networks in neonatal sepsis | 5 | -118.89 | 218/408 | GSE25504 |
| 77 | Effect of IL6 level on gene expression changes in Peripheral Blood Mononuclear cells (PBMC) induced by physical activity | 2 | -118.75 | 296/1092 | GSE12384 |
| 78 | The Hematopoietic System - Myeloid arm | 3 | -116.57 | 312/886 | GSE42519 |
| 79 | Expression data from severe asthmatics, mild asthmatics and healthy controls | 2 | -115.93 | 263/608 | GSE27011 |
| 80 | Effector cell signatures in peripheral blood after nasal allergen challenge: biomarkers of the allergic response | 1 | -115.66 | 504/2761 | GSE43497 |
| 81 | The human nose harbours a niche of olfactory ecto-mesenchymal stem cells displaying neurogenic and osteogenic properties | 2 | -114.8 | 321/1210 | GSE24598 |
| 82 | Gene Networks Specific for Innate Immunity Define Post-traumatic Stress Disorder [Affymetrix] | 2 | -114.79 | 435/1704 | GSE63878 |
| 83 | Expression data from Gambian children with and without the clinical signs of active trachoma: HG-focus array | 4 | -114.37 | 207/565 | GSE20430 |
| 84 | Effects of 30 days resveratrol supplementation on adipose tissue morphology and gene expression patterns in obese men | 2 | -114.25 | 292/945 | GSE42432 |
| 85 | Gene expression in M. tuberculosis and M. africanum infected tuberculosis patients prior to and following treatment | 3 | -114.06 | 215/495 | GSE62147 |
| 86 | Non-invasive Analysis of the Airway Transcriptome Discriminates Clinical Phenotypes of Asthma | 3 | -113.33 | 218/450 | GSE56396 |
| 87 | Effects of exercise on gene expression in human neutrophils | 1 | -113.31 | 542/2661 | GSE8668 |
| 88 | Gene expression profiles in white blood cells in response to exercise | 2 | -110.83 | 304/964 | GSE3606 |
| 89 | Whole Blood Cell Gene Expression Profiling in Patients with Coronary Artery Disease from the Cathgen Registry | 2 | -106.74 | 260/694 | GSE20680 |
| 90 | Comprehensive Study of Tobacco Smoke-Related Transcriptome Alterations in Maternal and Fetal Cells | 2 | -105.9 | 360/1459 | GSE27272 |
| 91 | A Brief Bout of Exercise Alters Gene Expression and Distinct Gene Pathways in PBMC of Early- and Late-Pubertal Females | 1 | -104.83 | 327/1301 | GSE14642 |
| 92 | Expression profiling of peripheral blood of chronic HCV infection | 8 | -103.93 | 156/225 | GSE59312 |
| 93 | Whole blood gene expression data from PFAPA syndrome | 5 | -103.52 | 192/382 | GSE17732 |
| 94 | Genome-wide expression kinetics of children with T1D-associated autoantibodies compared to healthy matched controls I | 2 | -103.25 | 186/387 | GSE30208 |
| 95 | Detection of cardiac allograft rejection and response to immunosuppressive therapy with peripheral blood gene expression | 3 | -101.6 | 229/620 | GSE5967 |
| 96 | Bone marrow gene expression of pediatric acute lymphoblastic leukemia (ALL) | 3 | -101.35 | 202/547 | GSE17703 |
| 97 | The Plasma Cell Signature in Autoimmune Disease (I) | 1 | -100.83 | 311/1005 | GSE45535 |
| 98 | The effect of listening to music on human transcriptome | 2 | -99.5 | 233/615 | GSE48624 |
| 99 | Expression of Neutrophil-related genes in patients with early sepsis-induced ARDS | 4 | -99.43 | 204/388 | GSE66890 |
| 100* | Whole Blood Gene Expression Profiles Distinguish Patients with Single versus Recurrent Venous Thromboembolism | 8 | -98.85 | 155/259 | GSE19151 |

* Only top 100 results are shown in the above table

^A^ GEO (Gene Expression Omnibus) accession number
