# Supplementary material for: Systems analysis-based assessment of post-treatment adverse events in lymphatic filariasis
Source: PLoS Negl Trop Dis. 2019 Sep 26;13(9):e0007697. doi: 10.1371/journal.pntd.0007697 (PMC6762072; doi:10.1371/journal.pntd.0007697)
Supplement: S8 Table — (DOCX) [file pntd.0007697.s013.docx]

**S8 Table. Variables in the random forest model (post-treatment fold change)**

| Variable | Average Mean Decrease in Accuracy |
| --- | --- |
| LPS binding protein (LBP) | 9.85 |
| Complement component 3 (C3) | -0.66 |
| Circulating immune complexes (CIC) | -2.35 |
| Complement Factor B (FB) | -2.92 |
| Circulating filarial antigen (CFA) | -4.20 |
| Complement component 4 (C4) | -5.01 |
